# GenSSI 2.0: multi-experiment structural identifiability analysis of SBML models

**DOI:** 10.1093/bioinformatics/btx735

**Published:** 2017-11-30

**Authors:** Thomas S Ligon, Fabian Fröhlich, Oana T Chiş, Julio R Banga, Eva Balsa-Canto, Jan Hasenauer

**Affiliations:** 1 Faculty of Physics and Center for NanoScience (CeNS), Ludwig-Maximilians-Universität, München, Germany; 2 Institute of Computational Biology, Helmholtz Zentrum München, München, Germany; 3 Center of Mathematics, Technische Universität München, München, Germany; 4 Technological Institute for Industrial Mathematics, University of Santiago de Compostela, Santiago de Compostela, Spain; 5 (Bio)Process Engineering Group, Spanish National Research Council, IIM-CSIC, Vigo, Spain

## Abstract

**Motivation:**

Mathematical modeling using ordinary differential equations is used in systems biology to improve the understanding of dynamic biological processes. The parameters of ordinary differential equation models are usually estimated from experimental data. To analyze a priori the uniqueness of the solution of the estimation problem, structural identifiability analysis methods have been developed.

**Results:**

We introduce GenSSI 2.0, an advancement of the software toolbox GenSSI (Generating Series for testing Structural Identifiability). GenSSI 2.0 is the first toolbox for structural identifiability analysis to implement Systems Biology Markup Language import, state/parameter transformations and multi-experiment structural identifiability analysis. In addition, GenSSI 2.0 supports a range of MATLAB versions and is computationally more efficient than its previous version, enabling the analysis of more complex models.

**Availability and implementation:**

GenSSI 2.0 is an open-source MATLAB toolbox and available at https://github.com/genssi-developer/GenSSI.

**Supplementary information:**

Supplementary data are available at *Bioinformatics* online.

## 1 Introduction

In systems biology, ordinary differential equation (ODE) models are used to describe the dynamics of biological processes such as gene regulation, signal transduction and metabolism. The development of these ODE models involves a series of steps, including the construction of the model and the estimation of the unknown model parameters, e.g. reaction rates. The individual steps pose fundamental challenges arising from properties of the model and the experimental design. One frequently encountered challenge in parameter estimation is the lack of structural identifiability. This means that some parameters cannot be uniquely determined even in the ideal scenario of continuous noise-free observations. Information about structural identifiability can be used to change the experimental design (observables or stimulation conditions) or to reformulate the model.

To facilitate structural identifiability analysis, multiple open-source software toolboxes have been developed, e.g. COMBOS (Meshkat *et al.*, 2014), DAISY (Bellu *et al.*, 2007), EAR (Anguelova *et al.*, 2012) and GenSSI (Chiş *et al.*, 2011a). These toolboxes provide a broad range of features and implement methods based on differential algebra, semi-numerical differential algebra, generating series and identifiability tableaus (see Supplementary Tables S1 and S2). However, the toolboxes do not support community standards, i.e. the Systems Biology Markup Language (SBML), and merely support individual experimental conditions. Both complicate the application in systems biology, where SBML models are usually used and experimental data for multiple experimental conditions are collected.

In this article, we introduce GenSSI 2.0, which provides an SBML import, automatic methods for multi-experiment structural identifiability analysis, and methods for the transformation of models. We provide a comparison of the currently available tools in the Supplementary Material and detailed documentation.

## 2 Features

**Fig. 1:**
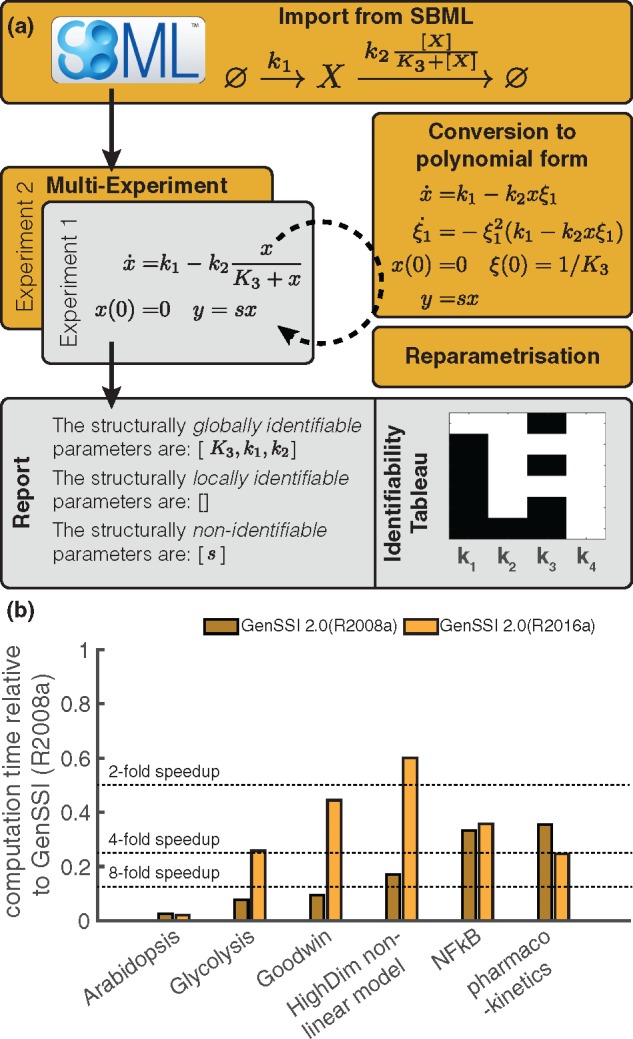
Properties of GenSSI 2.0. (**a**) Features implemented in GenSSI 2.0. Novel features are indicated in dark tone. (**b**) Performance evaluation of GenSSI and GenSSI 2.0 on different MATLAB versions

GenSSI 2.0 is a software toolbox for structural identifiability analysis of linear and non-linear ODE models (Fig. 1a). It couples the generating series approach with identifiability tableaus (Chiş *et al.*, 2011a). Using Lie derivatives of the ODE model, a system of equations is generated, the solvability properties of which provide information about global and local structural identifiability as well as non-identifiability (Chiş *et al.*, 2011b). The results are provided as text and visualized.

The implementation of GenSSI 2.0 is more efficient than that of earlier versions (Fig. 1b) and provides additional functionalities:

- An *import for the SBML models* is provided to facilitate the application of structural identifiability analysis in systems biology. This import exploits functionalities of the MATLAB toolbox SBMLimporter (https://github.com/ICB-DCM/SBMLimporter) and libSBML (Bornstein *et al.*, 2008).
- Routines for *multi-experiment structural identifiability analysis* are provided. These routines allow the analysis of structural identifiability if observation under multiple experimental conditions, e.g. different inputs or initial states, are available.
- Routines for *state and parameter transformations* are implemented to facilitate the removal of non-identifiable parameters and rescaling the variables. To accelerate the symbolic calculations, a routine for the automatic reformulations of ODEs with rational right-hand sides to ODEs with polynomial right-hand sides is provided (Ohtsuka, 2005).

A detailed description of all functionalities is included in the Reference Manual, which is available on GitHub.

GenSSI 2.0 has been tested on Windows, Mac and Linux with MATLAB version R2008a up to R2016a. To ensure long-term sustainability, the dependence on the Maple toolbox for symbolic math (only available for MATLAB 2010a or older) has been removed.

## 3 Application

The functionality of GenSSI 2.0 is illustrated on a series of benchmark problems included in the package, e.g. the Goodwin oscillator model and a model for mRNA transfection. We demonstrate the SBML import, the transformations and the importance of multi-experiment structural identifiability analysis. In addition, we performed a comparison with the earlier version of GenSSI, EAR, DAISY and COMBOS. This comparison revealed that GenSSI 2.0 is computationally more efficient than its precursor and that it provides more information than other tools (Fig. 1b).

## 4 Discussion and conclusion

In this study, we present GenSSI 2.0, a toolbox for structural identifiability analysis of models describing biological systems under multiple experimental conditions. GenSSI 2.0 is easy to use, i.e. it does not require advanced mathematical or programming skills, and provides an SBML import. The analysis provides information about the subsets/combinations of structurally identifiable parameters, which can be used to reformulate the model. To facilitate the development of GenSSI in a community effort, we provide an open-source publication on GitHub.

## Supplementary Material

Supplementary Data
