# Supplementary material for: GenSSI 2.0: multi-experiment structural identifiability analysis of SBML models
Source: Bioinformatics. 2017 Nov 30;34(8):1421–3. doi: 10.1093/bioinformatics/btx735 (PMC5905618; doi:10.1093/bioinformatics/btx735)
Supplement: Supplementary Data [file btx735_suppl_data.zip › Supplementary-Notes.pdf]

Supplementary Information to **GenSSI 2.0: Multi-experiment structural identifiability analysis of SBML models**

Thomas S. Ligon[1*], Fabian Fröhlich[2,3], Oana-Teodora Chiş[4], Julio R. Banga[5], Eva Balsa-Canto[5], Jan Hasenauer[2,3*]

[1]Faculty of Physics and Center for NanoScience (CeNS), Ludwig-Maximilians-Universität, 80539 München, Germany, [2]Institute of Computational Biology, Helmholtz Zentrum München, 85764 München, Germany, [3]Center of Mathematics, Technische Universität München, 85748 München, Germany, [4]Technological Institute for Industrial Mathematics, Campus Vida, Santiago de Compostela, Spain, [5](Bio)Process Engineering Group, Spanish National Research Council, IIM-CSIC, 36208 Vigo, Spain

*To whom correspondence should be addressed.

In this supplement, we provide a comparison of GenSSI 2.0 with the previous version and with alternative toolboxes for structural identifiability analysis. In addition, we present a collection of benchmark problems for structural identifiability analysis.

1 Toolboxes for Structural Identifiability Analysis

Over the last decade, a series of toolboxes for structural identifiability analysis have been developed. Among the well-established tools are COMBOS, DAISY, EAR and GenSSI 1.0. These toolboxes exploit different software bases, implement a range of approaches and provide different levels of information. An overview of the properties and features of the toolboxes is provided in Tables S1 and S2. The comparison in Table S2 reveals that GenSSI 2.0 provides the most comprehensive set of features.

2 Collection of Benchmark Problems

GenSSI 2.0 provides a comprehensive collection of benchmark problems for structural identifiability analysis. This collection contains models for metabolism, signal transduction and gene expression with a range of sizes and properties, e.g.:

- globally, locally and non-identifiable
- polynomial and rational kinetic laws
- with and without inputs
- single and multiple experimental conditions

The benchmark problems and their properties are provided in Table S3. This collection facilitates an evaluation of GenSSI 2.0 and a comparison with alternative software toolboxes for structural identifiability analysis.

3 Comparison of GenSSI 1.0 and 2.0

The implementation of GenSSI 2.0 is based on GenSSI 1.0, it has however been improved in several ways. To assess this improvement, we determined the runtimes of

- GenSSI 1.0 on MATLAB R2008a and
- GenSSI 2.0 on MATLAB R2008a and R20016b

for the individual benchmark problems. As GenSSI 1.0 relies on the Maple toolbox for symbolic math (only available for MATLAB 2010a or older), an evaluation of GenSSI 1.0 on MATLAB R20016a was not possible.

The comparison of the runtimes of GenSSI 1.0 and 2.0 on MATLAB R2008a revealed that GenSSI 2.0 is on average 10-times faster. For the Arabidopsis model, we even observe an acceleration of a factor of ~49. In addition to a reduction in the computation time, the memory requirements were slightly reduced.

While the improved efficiency of GenSSI 2.0 over GenSSI 1.0 was expected, the comparison of GenSSI 2.0 on different MATLAB versions led to surprising results. Even after several modifications, the runtime on MATLAB R2016b is on average 2-times higher than on MATLAB R2008a. This suggests that for the problem considered, the Maple toolbox for symbolic math included in previous versions of MATLAB is more efficient than the currently used symbolic engine (MuPAD). Nevertheless, GenSSI 2.0 on MATLAB R2016b is on average still >2-times faster than GenSSI 1.0 on MATLAB R2008a.

4 Comparison of GenSSI 2.0 and other Toolboxes for Structural Identifiability

To compare the different toolboxes for structural identifiability analysis, we used them to study the collection of benchmark problems. The implementations are COMBOS (version 1.5, November 2014), DAISY (version 1.9, 25 June 2014) and EAR (version 0.9.0, 3 July 2012). Table S5 summarizes the results of the comparison and reports problems which were encountered.

Remark: *We invested a substantial amount of effort in this comparison and tried to get all toolboxes running for all benchmark problems. Unfortunately, we encountered several difficulties. These difficulties might either be related to implementation errors on our side or problems of the respective tools.*

Our comparison indicates that GenSSI is the most flexible toolbox as it supports all the models considered. EAR supports most models, while DAISY and COMBOS provide limited support for models with parameter-dependent initial conditions. For models with parametric initial conditions, DAISY and COMBOS were run without these constraints, implying that the results are not necessary comparable. If applicable, EAR is computationally most efficient as it employs probabilistic differential algebra. GenSSI outperforms DAISY and COMBOS for most models; however, the efficiency of GenSSI as well as the results depend on the number of derivatives taken and on which parameters were considered. DAISY and COMBOS often fail to provide any results due to large computation times and memory requirements. The outputs of GenSSI are usually most informative as they include identifiability tableaus and explicit expressions for solutions to relations.

Table S1. Implementation and availability of different toolboxes for structural identifiability analysis.

	COMBOS	DAISY	EAR	GenSSI 1.0 and 2.0
**Method**	Differential algebra	Differential algebra	Semi-numerical probabilistic differential algebra	Generating series
**Software base**	Maxima	REDUCE	Mathematica	MATLAB
**Availability**	Web	Object Code	Object Code	Source Code
**Operating system**				
Windows		✓	✓	✓
Linux			✓	✓
Mac OS			✓	✓
Web	✓			
**URL**	http://biocyb1.cs.ucla.edu/combos	http://daisy.dei.unipd.it	http://www.fcc.chalmers.se/software/other-software/identifiabilityanalysis	https://github.com/genssi-developers/GenSSI
**Reference**	(Meshkat, et al., 2014)	(Bellu, et al., 2007)	(Anguelova, et al., 2012)	(Chiş, et al., 2011a)

Table S2. Features of structural identifiability toolboxes.

	COMBOS	DAISY	EAR	GenSSI 1.0	GenSSI 2.0
**Model and experimental setup**					
SBML import	×	×	×	×	✓
rational kinetic laws	×[1]	✓	✓	✓	✓
Parameter-dependent initial conditions	×[2]	×[2]	✓	✓	✓
Control inputs	✓	✓	✓	✓	✓
Multiple experimental conditions	×	×	×	×	✓
Transformation to polynomial form	×	×	×	×	✓
**Identifiability analysis**					
Local structural identifiability	✓	✓	✓	✓	✓
Global structural identifiability	✓	✓	×	✓	✓
Visualization of results	×	×	×	✓	✓
**Post-processing**					
State transformation	×	×	×	×	✓
Parameter transformation	×	×	×	×	✓

[1] COMBOS only supports polynomial kinetics, i.e. mass-action kinetics.

[2] COMBOS and DAISY do not support the usage of parameters that are not included in the vector field of the ODE model. Removing these parameters from our existing models changes them, making a comparison less meaningful.

Table S3. Collection of benchmark problems. The properties of all problems are reported along with some information about the biological process.

MODEL NAME	MODEL	VECTOR FIELD		INITIAL CONDITIONS	INPUT	EXPERIMENTAL CONDITIONS		IDENTIFIABILITY (as indicated by previous studies)			PROBLEM SIZE		REFERENCE
		poly-nomial	ratio-nal	parametrized	included	single	multiple	global	local	non	state variables	parame-ters	
`Arabidopsis`	Model for the circadian clock of Arabidopsis thaliana		✓		✓	✓		✓			7	27	(Locke, et al., 2005)
`ArabidopsisPoly`	circadian clock of Arabidopsis thaliana in polynomial form	✓		✓		✓		✓			16	27	this manuscript
`Bilirubin1`	4-component unidentifiable model for bilirubin kinetics	✓			✓	✓				✓	4	7	(Meshkat, et al., 2014)
`Bilirubin2`	4-component unidentifiable model with initial conditions for bilirubin kinetics	✓			✓	✓			✓		4	7	(Meshkat, et al., 2014)
`BIOMD03`	cell-cycle model		✓			✓					3	13	(Goldbeter, 1991)
`BIOMD10`	MAPK model		✓			✓					8	22	(Kholodenko, 2000)
`Cholesterol1`	2-component unidentifiable model for cholesterol turnover	✓			✓	✓				✓	2	5	(Meshkat, et al., 2014)
`Cholesterol2`	2-component identifiable model for cholesterol turnover	✓			✓	✓			✓		2	4	(Meshkat, et al., 2014)
`Degradation`	Model for catalyzed degradation		✓			✓				✓	1	3	this manuscript
`DegradationPoly`	Model for catalyzed degradation	✓		✓		✓				✓	2	3	this manuscript
`Glycolysis`	Model for glycolysis		✓	✓	✓	✓		✓			5	10	(Bartl, et al., 2010)
`Goodwin`	Model for a genetic oscillator		✓			✓			✓		3	8	(Goodwin, 1965)
`GoodwinPoly`	Model for a genetic oscillator in polynomial form	✓		✓		✓			✓		4	8	this manuscript
`HighDimNonLin`	Model for the evaluation of structural identifiability analysis methods		✓	✓	✓	✓		✓			20	22	(Saccomani, et al., 2010)

**MODEL NAME**	**MODEL**	**VECTOR FIELD**		**INITIAL CONDITIONS**	**INPUT**	**EXPERIMENTAL CONDITIONS**		**IDENTIFIABILITY** (as indicated by previous studies)			**PROBLEM SIZE**		**REFERENCE**
		poly-nomial	ratio-nal	parametrized	included	single	multiple	global	local	non	state variables	parame-ters	
`HIV`	4-component unidentifiable model for HIV	✓			✓	✓				✓	4	9	(Meshkat, et al., 2014)
`JAK-STAT`	Model for Epo-induced JAK-STAT signaling	✓		✓	✓	✓		✓			9	7	(Raue, et al., 2009)
`NFkB`	Model for signal transduc-tion for the nuclear factor (NF) κB	✓		✓	✓	✓				✓	15	27	(Lipniacki, et al., 2004)
`NGF-Erk`	Model for NGF induced Erk signaling		✓	✓		✓					5	15	(Hasenauer, et al., 2014)
`Pharmacokinetics`	Pharmacokinetic model for the ligands of the macro-phage mannose receptor		✓	✓		✓			✓		4	9	(Demignot and Domurado, 1987)
`Pharmacokinetics Poly`	`Pharmacokinetics` model in polynomial form	✓		✓		✓			✓		5	9	this manuscript
`Thyroid1`	3-component unidentifiable model for thyroid kinetics	✓			✓	✓				✓	3	7	(Meshkat, et al., 2014)
`Thyroid2`	3-component unidentifiable model with initial conditions for thyroid kinetics	✓			✓	✓				✓	3	7	(Meshkat, et al., 2014)
`Transfection_2State`	Model for mRNA transfec-tion	✓		✓	✓	✓				✓	2	4	(Leonhardt, et al., 2013)
`Transfection_2State _MultiEx`	Multi-experiment model for mRNA transfection	✓		✓	✓		✓			✓	4	6	this manuscript (reference manual)
`Transfection_4State`	Model for mRNA transfec-tion, enzyme degradation	✓		✓		✓			✓		4	7	(Leonhardt, et al., 2013)
`Transfection_4State _Transformation`	Transformation model for mRNA transfection	✓		✓		✓					8	4	this manuscript (reference manual)

Table S4. Performance comparison of GenSSI 1.0 and 2.0. The runtime GenSSI 1.0 under MATLAB R2008a as well as the runtime of runtime GenSSI 2.0 under MATLAB R2008a and R2016b is reported in seconds. GenSSI 1.0 does not run under MATLAB versions 2008b and newer. The computation was performed on a PC with an Intel i7-6700 CPU (3.4 GHz, 4 cores, 8 threads) and 16GB of RAM running MATLAB R2016b with Symbolic Math Toolbox version 7.1 and MATLAB R2008a with Symbolic Math Toolbox version 3.2.3.

MODEL NAME	GenSSI 1.0		GenSSI 2.0	
	on R2008a	on R2016b	on R2008a	on R2017a
`Arabidopsis`	348.4	-	8.8	5.4
`Glycolysis`	19.4	-	1.5	14.2
`Goodwin`	6.3	-	0.6	8.0
`HighDimNonLin`	7.0	-	1.2	8.8
`NFkB`	8.7	-	2.9	9.8
`Pharmacokinetics*`	6.5	-	2.3	2.2

**The runtime was measured using two Lie derivatives, which works for both versions. Increasing the number to four Lie derivatives yields a higher rank and runs in 560.7 seconds on V2/R20017a, but fails to complete for GenSSI 1.0 and 2.0 under R2008a.*

Table S5. Comparison of different toolboxes for structural identifiability analysis using the collection of benchmark problems. For each combination of model and toolbox, three properties are reported. The first row indicates if a model was not tested (-), a model was not supported by the toolbox (~), a model was supported by the toolbox but the calculations did not complete successfully (✗), and a model was supported by the toolbox and the calculations completed successfully (✓). If the calculations completed successfully, the second row indicates the results of the identifiability analysis: non-identifiable; partially identifiable (meaning that some but not all parameters are identifiable); locally identifiable; and globally identifiable. The third row indicates the time required for the calculations. All calculations were performed on a PC with an Intel i7-6700 CPU (3.4 GHz, 4 cores, 8 threads) and 16GB of RAM. For model with parameterized initial conditions, COMBOS and DAISY indicate the results without these additional constraints. The different toolboxes provide outputs with different levels of information, e.g., DAISY does not provide information about partial identifiability.

	COMBOS	DAISY	EAR	GenSSI 2.0
`Arabidopsis`	~	✗	✓<br>partially identifiable<br>9.3	✓<br>globally identifiable<br>5.4
`ArabidopsisPoly`	~	✗	-	✓<br>globally identifiable<br>6.0
`Bilirubin1`	✓<br>partially identifiable[3]<br>14.8	✓<br>non-identifiable<br>1.0	✓<br>partially identifiable<br>0.4	✓<br>partially identifiable[2]<br>2.4
`Bilirubin2`	✓<br>identifiable<br>15.8	✓<br>non-identifiable<br>1.0	✓<br>(locally) identifiable<br>0.5	✓<br>locally identifiable<br>17,767.0
`BIOMD0000000003`	✓<br>identifiable<br>5.8	✓<br>non-identifiable<br>1.0	✓<br>(locally) identifiable<br>0.4	✓<br>globally identifiable<br>19.1
`BIOMD0000000010`	~	~	~	✓<br>globally identifiable<br>23.3
`Cholesterol1`	✓<br>partially identifiable<br>1.5	✓<br>non-identifiable<br>1.0	✓<br>partially identifiable<br>0.2	✓<br>partially identifiable[2]<br>5.3
`Cholesterol2`	✓<br>identifiable<br>0.4	✓<br>non-identifiable<br>1.0	✓<br>(locally) identifiable<br>0.2	✓<br>globally identifiable<br>7.3
`Degradation`	✓<br>identifiable<br>0.4	✓<br>globally identifiable<br>1.0	✓<br>(locally) identifiable<br>0.2	✓<br>globally identifiable<br>6.4
`DegradationPoly`	~[1]<br>partially identifiable[3]<br>0.7	~[1]<br>globally identifiable<br>1.0	✓<br>(locally) identifiable<br>0.2	✓<br>globally identifiable<br>6.6
`Glycolysis`	~[1]	~[1]<br>globally identifiable<br>3.0	✓<br>(locally) identifiable<br>0.7	✓<br>globally identifiable<br>14.2

	COMBOS	DAISY	EAR	GenSSI 2.0
Goodwin	~	✓<br>non-identifiable<br>1.0	~[3]<br>partially identifiable<br>0.5	✓<br>locally identifiable<br>8.0
GoodwinPoly	~[1]	~[1]<br>non-identifiable<br>1.0	-	✓<br>locally identifiable<br>9.1
HighDimNonLin	~[1]	~[1]<br>globally identifiable<br>58.0	✓<br>(locally) identifiable<br>2.3	✓<br>globally identifiable<br>8.8
HIV	✓<br>partially identifiable[3]<br>35.9	✓<br>non-identifiable<br>1.2	✓<br>partially identifiable<br>0.3	✓<br>non-identifiable<br>2.8
JAK-STAT	~[1]	~[1]<br>non-identifiable<br>2.0	✓<br>partially identifiable<br>1.3	✓<br>non-identifiable<br>21.2
NFkB	~[1]	~[1]<br>non-identifiable<br>12.1	✓<br>non-identifiable<br>17.9	✓<br>partially identifiable<br>9.8
NGF-Erk	~[1]	~[1]<br>non-identifiable<br>1.0	~[3]<br>non-identifiable<br>80.4	✓<br>locally identifiable<br>256.6
Pharmacokinetics	×[1]	~[1]<br>globally identifiable<br>3.0	✓<br>(locally) identifiable<br>0.7	✓<br>globally identifiable<br>539.0
PharmacokineticsPoly	×[1]	~[1]<br>non-identifiable<br>843.6	✓<br>(locally) identifiable<br>0.9	✓<br>locally identifiable<br>276.5
Thyroid1	✓<br>partially identifiable<br>23.3	✓<br>non-identifiable<br>1.0	✓<br>partially identifiable<br>0.4	✓<br>locally identifiable[2]<br>1069.8
Thyroid2	✓<br>partially identifiable<br>23.2	✓<br>non-identifiable<br>1.0	✓<br>partially identifiable<br>0.4	✓<br>partially identifiable[2]<br>56.7
Transfection_2State	~[1]<br>partially identifiable<br>1.2	~[1]<br>non-identifiable<br>1.1	✓<br>partially identifiable<br>0.2	✓<br>partially identifiable[2]<br>11.8
Transfection_2State_ MultiExp	×	~[1]<br>non-identifiable<br>1.0	✓<br>partially identifiable<br>0.8	✓<br>partially identifiable[2]<br>11.8
Transfection_4State	~[1]<br>partially identifiable<br>34.8	~[1]<br>globally identifiable<br>1.0	✓<br>partially identifiable<br>2.1	✓<br>partially identifiable[2]<br>46.6
Transfection_4State_ Transformation	~[1]<br>partially identifiable<br>11.4	~[1]<br>non-identifiable<br>1.0	✓<br>partially identifiable<br>8.6	✓<br>locally identifiable[2]<br>667.0

[1] *Parameterization of the initial conditions were disregarded in order to obtain some results. In this case a comparison is not possible.*

[2] *GenSSI reported that the Jacobian did not have full rank, implying that only a subset of the parameters can be identified.*

[3] *Model was reformulated to avoid the parameter in the exponential function (which is not supported by EAR).*

5 References

Anguelova, M., Karlsson, J. and Jirstrand, M. (2012) Minimal output sets for identifiability, *Mathematical Biosciences*, 239, 139-153.

Bartl, M., et al. (2010) Just-in-time activation of a glycolysis inspired metabolic network-solution with a dynamic optimization approach. *Proceedings 55nd International Scientific Colloquium*.

Bellu, G., et al. (2007) DAISY: a new software tool to test global identifiability of biological and physiological systems, *Computer Methods and Programs in Biomedicine*, 88, 52-61.

Chiş, O., Banga, J.R. and Balsa-Canto, E. (2011a) GenSSI: a software toolbox for structural identifiability analysis of biological models, *Bioinformatics*, 27, 2610-2611.

Demignot, S. and Domurado, D. (1987) Effect of prosthetic sugar groups on the pharmacokinetics of glucose-oxidase, *Drug Design and Delivery*, 1, 333-348.

Goldbeter, A. (1991) A minimal cascade model for the mitotic oscillator involving cyclin and cdc2 kinase, *Proceedings of the National Academy of Sciences of the United States of America*, 88, 9107-9111.

Goodwin, B.C. (1965) Oscillatory behavior in enzymatic control processes, *Advances in Enzyme Regulation*, 3, 425-437.

Hasenauer, J., et al. (2014) ODE constrained mixture modelling: a method for unraveling subpopulation structures and dynamics, *PLOS Computational Biology*, 10, e1003686.

Kholodenko, B.N. (2000) Negative feedback and ultrasensitivity can bring about oscillations in the mitogen-activated protein kinase cascades, *European Journal of Biochemistry*, 267, 1583-1588.

Leonhardt, C., et al. (2013) Single-cell mRNA transfection studies: delivery, kinetics and statistics by numbers, *Nanomedicine: Nanotechnology, Biology and Medicine*.

Lipniacki, T., et al. (2004) Mathematical model of NF-κB regulatory module, *Journal of Theoretical Biology*, 228, 195-215.

Locke, J.C.W., Millar, A.J. and Turner, M.S. (2005) Modelling genetic networks with noisy and varied experimental data: the circadian clock in Arabidopsis thaliana, *Journal of Theoretical Biology*, 234, 383-393.

Meshkat, N., Kuo, C.E.-z. and DiStefano, J., III (2014) On finding and using identifiable parameter combinations in nonlinear dynamic Systems Biology models and COMBOS: a novel Web implementation, *PLoS ONE*, 9, e110261.

Raue, A., et al. (2009) Structural and practical identifiability analysis of partially observed dynamical models by exploiting the profile likelihood, *Bioinformatics*, 25, 1923-1929.

Saccomani, M.P., et al. (2010) Examples of testing global identifiability of biological and biomedical models with the DAISY software, *Computers in Biology and Medicine*, 40, 402-407.
