# Supplementary material for: GenSSI 2.0: multi-experiment structural identifiability analysis of SBML models
Source: Bioinformatics. 2017 Nov 30;34(8):1421–3. doi: 10.1093/bioinformatics/btx735 (PMC5905618; doi:10.1093/bioinformatics/btx735)
Supplement: Supplementary Data [file btx735_suppl_data.zip › EAR.zip!/EAR/Arabidopsis.pdf]

```
Needs["IdentifiabilityAnalysis`"]
startTime = AbsoluteTime[]
```

$3.6875441632555340 \times 10^9$

```
vars = {x1, x2, x3, x4, x5, x6, x7}
```

{x1, x2, x3, x4, x5, x6, x7}

```
params = {p1, p2, p3, p4, p5, p6, p7, p8, p9, p10, p11, p12, p13,
   p14, p15, p16, p17, p18, p19, p20, p21, p22, p23, p24, p25, p26, p27}
```

{p1, p2, p3, p4, p5, p6, p7, p8, p9, p10, p11, p12, p13, p14, p15, p16, p17, p18, p19, p20, p21, p22, p23, p24, p25, p26, p27}

```
sys =
 {x1'[t] == p1 * x6[t] / (p3 + x6[t]) - p5 * x1[t] / (p12 + x1[t]) + p26 * x7[t] * u1[t],
  x2'[t] == p19 * x1[t] - p22 * x2[t] + p23 * x3[t] - p6 * x2[t] / (p13 + x2[t]),
  x3'[t] == p22 * x2[t] - p23 * x2[t] - p7 * x3[t] / (p14 + x3[t]),
  x4'[t] == p2 * p4^2 / (p4^2 + x3[t]^2) - p8 * x4[t] / (p15 + x4[t]),
  x5'[t] ==  p20 * x4[t] - p24 * x5[t] + p25 * x6[t] - p9 * x6[t] / (p16 + x5[t]),
  x6'[t] == p24 * x5[t] - p25 * x6[t] - p10 * x6[t] / (p17 + x6[t]),
  x7'[t] == p21 - p11 * x7[t] / (p18 + x7[t]) - (p21 + p27 * x7[t]) * u2[t],
  x1[0] == 0, x2[0] == 0, x3[0] == 0, x4[0] == 0, x5[0] == 0,  x6[0] == 0,  x7[0] == 0}
```

$$\left\{x1'[t] = -\frac{p5\, x1[t]}{p12 + x1[t]} + \frac{p1\, x6[t]}{p3 + x6[t]} + p26\, u1[t]\, x7[t],\right.$$

$$x2'[t] = p19\, x1[t] - p22\, x2[t] - \frac{p6\, x2[t]}{p13 + x2[t]} + p23\, x3[t],$$

$$x3'[t] = p22\, x2[t] - p23\, x2[t] - \frac{p7\, x3[t]}{p14 + x3[t]},\ x4'[t] = \frac{p2\, p4^2}{p4^2 + x3[t]^2} - \frac{p8\, x4[t]}{p15 + x4[t]},$$

$$x5'[t] = p20\, x4[t] - p24\, x5[t] + p25\, x6[t] - \frac{p9\, x6[t]}{p16 + x5[t]},$$

$$x6'[t] = p24\, x5[t] - p25\, x6[t] - \frac{p10\, x6[t]}{p17 + x6[t]},$$

$$x7'[t] = p21 - \frac{p11\, x7[t]}{p18 + x7[t]} - u2[t]\,(p21 + p27\, x7[t]),\ x1[0] = 0,$$

$$\left. x2[0] = 0,\ x3[0] = 0,\ x4[0] = 0,\ x5[0] = 0,\ x6[0] = 0,\ x7[0] = 0\right\}$$

```
output = {x1[t], x4[t]}
```

{x1[t], x4[t]}

```
iad = IdentifiabilityAnalysis[{sys, output}, vars, params, t, {u1, u2}]
```

IdentifiabilityAnalysisData[False, <>]

```
iad["IdentifiableQ"]
```

False

```
iad["DegreesOfFreedom"]
```

3

```
iad["NonIdentifiableParameters"]
```

{p10, p11, p13, p14, p16, p17, p18, p19, p20, p21, p26, p3, p4, p6, p7, p9}

```
endTime = AbsoluteTime[]
N[endTime - startTime]
```

$3.6875441725425615 \times 10^{9}$

9.28703
