# Supplementary material for: GenSSI 2.0: multi-experiment structural identifiability analysis of SBML models
Source: Bioinformatics. 2017 Nov 30;34(8):1421–3. doi: 10.1093/bioinformatics/btx735 (PMC5905618; doi:10.1093/bioinformatics/btx735)
Supplement: Supplementary Data [file btx735_suppl_data.zip › EAR.zip!/EAR/Bilirubin1.pdf]

```
In[1]:= Needs["IdentifiabilityAnalysis`"]
        startTime = AbsoluteTime[]

Out[2]= 3.6879518348563880 × 10^9

In[3]:= vars = {x1, x2, x3, x4}

Out[3]= {x1, x2, x3, x4}

In[4]:= params = {k03, k04, k13, k24, k31, k42, k43}

Out[4]= {k03, k04, k13, k24, k31, k42, k43}

In[5]:= sys = {x1 '[t] == -k31 * x1[t] + k13 * x3[t] + u1[t],
          x2 '[t] == -k42 * x2[t] + k24 * x4[t],
          x3 '[t] == k31 * x1[t] - (k03 + k13 + k43) * x3[t],
          x4 '[t] == k42 * x2[t] + k43 * x3[t] - (k04 + k24) * x4[t],
          x1[0] == 0, x2[0] == 0, x3[0] == 0, x4[0] == 0}

Out[5]= {x1′[t] == u1[t] - k31 x1[t] + k13 x3[t],
         x2′[t] == -k42 x2[t] + k24 x4[t], x3′[t] == k31 x1[t] - (k03 + k13 + k43) x3[t],
         x4′[t] == k42 x2[t] + k43 x3[t] - (k04 + k24) x4[t],
         x1[0] == 0, x2[0] == 0, x3[0] == 0, x4[0] == 0}

In[6]:= output = {x1[t], x2[t]}

Out[6]= {x1[t], x2[t]}

In[7]:= iad = IdentifiabilityAnalysis[{sys, output}, vars, params, t, {u1}]

Out[7]= IdentifiabilityAnalysisData[False, <>]

In[8]:= iad["IdentifiableQ"]

Out[8]= False

In[9]:= iad["DegreesOfFreedom"]

Out[9]= 1

In[10]:= iad["NonIdentifiableParameters"]

Out[10]= {k03, k04, k24, k42, k43}

In[11]:= endTime = AbsoluteTime[]
         N[endTime - startTime]

Out[11]= 3.6879518353041970 × 10^9

Out[12]= 0.447809
```
