# Supplementary material for: GenSSI 2.0: multi-experiment structural identifiability analysis of SBML models
Source: Bioinformatics. 2017 Nov 30;34(8):1421–3. doi: 10.1093/bioinformatics/btx735 (PMC5905618; doi:10.1093/bioinformatics/btx735)
Supplement: Supplementary Data [file btx735_suppl_data.zip › EAR.zip!/EAR/Bilirubin2.pdf]

In[62]:=
```
Needs["IdentifiabilityAnalysis`"]
startTime = AbsoluteTime[]
```

Out[63]= $3.6877993648789118 \times 10^9$

In[64]:=
```
vars = {x1, x2, x3, x4}
```

Out[64]= {x1, x2, x3, x4}

In[65]:=
```
params = {k01, k12, k13, k14, k21, k31, k41}
```

Out[65]= {k01, k12, k13, k14, k21, k31, k41}

In[66]:=
```
sys = {x1'[t] ==
    - (k21 + k31 + k41 + k01) * x1[t] + k12 * x2[t] + k13 * x3[t] + k14 * x4[t] + u1[t],
   x2'[t] == k21 * x1[t] - k12 * x2[t],
   x3'[t] == k31 * x1[t] - k13 * x3[t],
   x4'[t] == k41 * x1[t] - k14 * x4[t],
   x1[0] == 0, x2[0] == 0, x3[0] == 0, x4[0] == 0}
```

Out[66]=
```
{x1′[t] == u1[t] + (-k01 - k21 - k31 - k41) x1[t] + k12 x2[t] + k13 x3[t] + k14 x4[t],
 x2′[t] == k21 x1[t] - k12 x2[t], x3′[t] == k31 x1[t] - k13 x3[t],
 x4′[t] == k41 x1[t] - k14 x4[t], x1[0] == 0, x2[0] == 0, x3[0] == 0, x4[0] == 0}
```

In[67]:=
```
output = {x1[t]}
```

Out[67]= {x1[t]}

In[68]:=
```
iad = IdentifiabilityAnalysis[{sys, output}, vars, params, t, {u1}]
```

Out[68]= IdentifiabilityAnalysisData[True, <>]

In[69]:=
```
iad["IdentifiableQ"]
```

Out[69]= True

In[70]:=
```
iad["DegreesOfFreedom"]
```

Out[70]= 0

In[71]:=
```
iad["NonIdentifiableParameters"]
```

Out[71]= {}

In[72]:=
```
endTime = AbsoluteTime[]
N[endTime - startTime]
```

Out[72]= $3.6877993653688264 \times 10^9$

Out[73]= 0.489915
