# Supplementary material for: GenSSI 2.0: multi-experiment structural identifiability analysis of SBML models
Source: Bioinformatics. 2017 Nov 30;34(8):1421–3. doi: 10.1093/bioinformatics/btx735 (PMC5905618; doi:10.1093/bioinformatics/btx735)
Supplement: Supplementary Data [file btx735_suppl_data.zip › EAR.zip!/EAR/BIOMD0000000003.pdf]

```
In[1]:= Needs["IdentifiabilityAnalysis`"]
        startTime = AbsoluteTime[]
        vars = {x1, x2, x3}
        params = {p1, p2, p3, p4, p5, p6, p7, p8, p9, p10, p11, p12, p13}
        sys = {x1'[t] == p4 - x1[t] * p5 - x1[t] * x3[t] * p6 * (x1[t] + p7) ^ - 1,
           x2'[t] == - x2[t] * p9 * (p10 + x2[t]) ^ - 1 -
              x1[t] * p1 * (x1[t] + p3) ^ - 1 * (x2[t] - 1) * (p8 - x2[t] + 1) ^ - 1,
           x3'[t] == - p13 * x3[t] * (p12 + x3[t]) ^ - 1 -
              x2[t] * p2 * (x3[t] - 1) * (p11 - x3[t] + 1) ^ - 1,
           x1[0] == 1 / 100, x2[0] == 1 / 100, x3[0] == 1 / 100}
        output = {x1[t], x2[t], x3[t]}
```

Out[2]= $3.7143134861583132 \times 10^{9}$

Out[3]= {x1, x2, x3}

Out[4]= {p1, p2, p3, p4, p5, p6, p7, p8, p9, p10, p11, p12, p13}

Out[5]=
$$\left\{x1'[t] == p4 - p5\, x1[t] - \frac{p6\, x1[t]\, x3[t]}{p7 + x1[t]},\right.$$
$$x2'[t] == -\frac{p1\, x1[t]\, (-1 + x2[t])}{(p3 + x1[t])\, (1 + p8 - x2[t])} - \frac{p9\, x2[t]}{p10 + x2[t]},$$
$$\left. x3'[t] == -\frac{p2\, x2[t]\, (-1 + x3[t])}{1 + p11 - x3[t]} - \frac{p13\, x3[t]}{p12 + x3[t]},\ x1[0] == \frac{1}{100},\ x2[0] == \frac{1}{100},\ x3[0] == \frac{1}{100}\right\}$$

Out[6]= {x1[t], x2[t], x3[t]}

```
In[7]:= iad = IdentifiabilityAnalysis[{sys, output}, vars, params, t]
```

Out[7]= IdentifiabilityAnalysisData[True, <>]

```
In[8]:= iad["IdentifiableQ"]
```

Out[8]= True

```
In[9]:= iad["DegreesOfFreedom"]
```

Out[9]= 0

```
In[10]:= iad["NonIdentifiableParameters"]
```

Out[10]= { }

```
In[11]:= endTime = AbsoluteTime[]
         N[endTime - startTime]
```

Out[11]= $3.7143134865432848 \times 10^{9}$

Out[12]= 0.384972
