# Supplementary material for: GenSSI 2.0: multi-experiment structural identifiability analysis of SBML models
Source: Bioinformatics. 2017 Nov 30;34(8):1421–3. doi: 10.1093/bioinformatics/btx735 (PMC5905618; doi:10.1093/bioinformatics/btx735)
Supplement: Supplementary Data [file btx735_suppl_data.zip › EAR.zip!/EAR/Cholesterol1.pdf]

```
In[98]:= Needs["IdentifiabilityAnalysis`"]
         startTime = AbsoluteTime[]

Out[99]= 3.6877999395700907 × 10^9

In[100]:= vars = {x1, x2}

Out[100]= {x1, x2}

In[101]:= params = {k01, k02, k12, k21, V1}

Out[101]= {k01, k02, k12, k21, V1}

In[102]:= sys = {x1'[t] == k12 * x2[t] - (k01 + k21) * x1[t] + u1[t],
             x2'[t] == k21 * x1[t] - (k02 + k12) * x2[t],
             x1[0] == 0, x2[0] == 0}

Out[102]= {x1′[t] == u1[t] - (k01 + k21) x1[t] + k12 x2[t],
           x2′[t] == k21 x1[t] - (k02 + k12) x2[t], x1[0] == 0, x2[0] == 0}

In[103]:= output = {x1[t] / V1}

Out[103]= {x1[t]/V1}

In[104]:= iad = IdentifiabilityAnalysis[{sys, output}, vars, params, t, {u1}]

Out[104]= IdentifiabilityAnalysisData[False, <>]

In[105]:= iad["IdentifiableQ"]

Out[105]= False

In[106]:= iad["DegreesOfFreedom"]

Out[106]= 1

In[107]:= iad["NonIdentifiableParameters"]

Out[107]= {k01, k02, k12, k21}

In[108]:= endTime = AbsoluteTime[]
          N[endTime - startTime]

Out[108]= 3.6877999397862439 × 10^9

Out[109]= 0.216153
```
