# Supplementary material for: GenSSI 2.0: multi-experiment structural identifiability analysis of SBML models
Source: Bioinformatics. 2017 Nov 30;34(8):1421–3. doi: 10.1093/bioinformatics/btx735 (PMC5905618; doi:10.1093/bioinformatics/btx735)
Supplement: Supplementary Data [file btx735_suppl_data.zip › EAR.zip!/EAR/Cholesterol2.pdf]

```
In[110]:= Needs["IdentifiabilityAnalysis`"]
          startTime = AbsoluteTime[]

Out[111]= 3.6878000820094578 × 10^9

In[112]:= vars = {x1, x2}

Out[112]= {x1, x2}

In[113]:= params = {k02, k12, k21, V1}

Out[113]= {k02, k12, k21, V1}

In[114]:= sys = {x1'[t] == k12 * x2[t] - (1 + k21) * x1[t] + u1[t],
            x2'[t] == k21 * x1[t] - (k02 + k12) * x2[t],
            x1[0] == 0, x2[0] == 0}

Out[114]= {x1′[t] == u1[t] - (1 + k21) x1[t] + k12 x2[t],
           x2′[t] == k21 x1[t] - (k02 + k12) x2[t], x1[0] == 0, x2[0] == 0}

In[115]:= output = {x1[t] / V1}
```

Out[115]= $\left\{\frac{x1[t]}{V1}\right\}$

```
In[116]:= iad = IdentifiabilityAnalysis[{sys, output}, vars, params, t, {u1}]

Out[116]= IdentifiabilityAnalysisData[True, <>]

In[117]:= iad["IdentifiableQ"]

Out[117]= True

In[118]:= iad["DegreesOfFreedom"]

Out[118]= 0

In[119]:= iad["NonIdentifiableParameters"]

Out[119]= {}

In[120]:= endTime = AbsoluteTime[]
          N[endTime - startTime]

Out[120]= 3.6878000821939887 × 10^9

Out[121]= 0.184531
```
