# Supplementary material for: GenSSI 2.0: multi-experiment structural identifiability analysis of SBML models
Source: Bioinformatics. 2017 Nov 30;34(8):1421–3. doi: 10.1093/bioinformatics/btx735 (PMC5905618; doi:10.1093/bioinformatics/btx735)
Supplement: Supplementary Data [file btx735_suppl_data.zip › EAR.zip!/EAR/Degradation.pdf]

```
In[146]:= Needs["IdentifiabilityAnalysis`"]
          startTime = AbsoluteTime[]

Out[147]= 3.6878004675512553 × 10^9

In[148]:= vars = {x1}

Out[148]= {x1}

In[149]:= params = {k1, k2, k3}

Out[149]= {k1, k2, k3}

In[150]:= sys = {x1'[t] == k1 - k2 * x1[t] / (k3 + x1[t]),
            x1[0] == 0}
```

Out[150]= $\left\{x1'[t] == k1 - \frac{k2\,x1[t]}{k3 + x1[t]},\ x1[0] == 0\right\}$

```
In[151]:= output = {x1[t]}

Out[151]= {x1[t]}

In[152]:= iad = IdentifiabilityAnalysis[{sys, output}, vars, params, t, {u1}]

Out[152]= IdentifiabilityAnalysisData[True, <>]

In[153]:= iad["IdentifiableQ"]

Out[153]= True

In[154]:= iad["DegreesOfFreedom"]

Out[154]= 0

In[155]:= iad["NonIdentifiableParameters"]

Out[155]= {}

In[156]:= endTime = AbsoluteTime[]
          N[endTime - startTime]

Out[156]= 3.6878004677049334 × 10^9

Out[157]= 0.153678
```
