# Supplementary material for: GenSSI 2.0: multi-experiment structural identifiability analysis of SBML models
Source: Bioinformatics. 2017 Nov 30;34(8):1421–3. doi: 10.1093/bioinformatics/btx735 (PMC5905618; doi:10.1093/bioinformatics/btx735)
Supplement: Supplementary Data [file btx735_suppl_data.zip › EAR.zip!/EAR/DegradationPoly.pdf]

```
In[134]:= Needs["IdentifiabilityAnalysis`"]
          startTime = AbsoluteTime[]

Out[135]= 3.6878003580970291 × 10^9

In[136]:= vars = {x1, x2}

Out[136]= {x1, x2}

In[137]:= params = {k1, k2, k3}

Out[137]= {k1, k2, k3}

In[138]:= sys = {x1'[t] == k1 - k2 * x1[t] * x2[t],
             x2'[t] == -x2[t]^2 * (k1 - k2 * x1[t] * x2[t]),
             x1[0] == 0, x2[0] == 1 / k3}

Out[138]= {x1'[t] == k1 - k2 x1[t] x2[t],
           x2'[t] == -x2[t]^2 (k1 - k2 x1[t] x2[t]), x1[0] == 0, x2[0] == 1/k3}

In[139]:= output = {x1[t]}

Out[139]= {x1[t]}

In[140]:= iad = IdentifiabilityAnalysis[{sys, output}, vars, params, t, {u1}]

Out[140]= IdentifiabilityAnalysisData[True, <>]

In[141]:= iad["IdentifiableQ"]

Out[141]= True

In[142]:= iad["DegreesOfFreedom"]

Out[142]= 0

In[143]:= iad["NonIdentifiableParameters"]

Out[143]= {}

In[144]:= endTime = AbsoluteTime[]
          N[endTime - startTime]

Out[144]= 3.6878003582973923 × 10^9

Out[145]= 0.200363
```
