# Supplementary material for: GenSSI 2.0: multi-experiment structural identifiability analysis of SBML models
Source: Bioinformatics. 2017 Nov 30;34(8):1421–3. doi: 10.1093/bioinformatics/btx735 (PMC5905618; doi:10.1093/bioinformatics/btx735)
Supplement: Supplementary Data [file btx735_suppl_data.zip › EAR.zip!/EAR/Glycolysis.pdf]

```
In[1]:= Needs["IdentifiabilityAnalysis`"]
        startTime = AbsoluteTime[]

Out[2]= 3.6879518830351640 × 10^9

In[3]:= vars = {x1, x2, x3, x4, x5}

Out[3]= {x1, x2, x3, x4, x5}

In[4]:= params = {k1, k2, k3, k4, kM, S1, S2, S3, S4, S5}

Out[4]= {k1, k2, k3, k4, kM, S1, S2, S3, S4, S5}

In[5]:= sys = {x1'[t] == -k1 * x1[t] * u1[t] / (x1[t] + kM),
          x2'[t] == k1 * x1[t] * u1[t] / (x1[t] + kM) - k2 * x2[t] * u2[t] / (x2[t] + kM),
          x3'[t] == k2 * x2[t] * u2[t] / (x2[t] + kM) - k3 * x3[t] * u3[t] / (x3[t] + kM),
          x4'[t] == k2 * x2[t] * u2[t] / (x2[t] + kM) +
            k3 * x3[t] * u3[t] / (x3[t] + kM) - k4 * x4[t] * u4[t] / (x4[t] + kM),
          x5'[t] ==  k4 * x4[t] * u5[t] / (x4[t] + kM),
          x1[0] == S1, x2[0] == S2, x3[0] == S3, x4[0] == S4, x5[0] == S5}
```

Out[5]=
$$\left\{x1'[t] == -\frac{k1\,u1[t]\,x1[t]}{kM + x1[t]},\ x2'[t] == \frac{k1\,u1[t]\,x1[t]}{kM + x1[t]} - \frac{k2\,u2[t]\,x2[t]}{kM + x2[t]},\right.$$
$$x3'[t] == \frac{k2\,u2[t]\,x2[t]}{kM + x2[t]} - \frac{k3\,u3[t]\,x3[t]}{kM + x3[t]},$$
$$x4'[t] == \frac{k2\,u2[t]\,x2[t]}{kM + x2[t]} + \frac{k3\,u3[t]\,x3[t]}{kM + x3[t]} - \frac{k4\,u4[t]\,x4[t]}{kM + x4[t]},\ x5'[t] == \frac{k4\,u5[t]\,x4[t]}{kM + x4[t]},$$
$$\left. x1[0] == S1,\ x2[0] == S2,\ x3[0] == S3,\ x4[0] == S4,\ x5[0] == S5\right\}$$

```
In[6]:= controls = {u1, u2, u3, u4, u5}

Out[6]= {u1, u2, u3, u4, u5}

In[7]:= output = {x1[t], x2[t], x3[t], x4[t], x5[t]}

Out[7]= {x1[t], x2[t], x3[t], x4[t], x5[t]}

In[8]:= iad = IdentifiabilityAnalysis[{sys, output}, vars, params, t, controls]

Out[8]= IdentifiabilityAnalysisData[True, <>]

In[9]:= iad["IdentifiableQ"]

Out[9]= True

In[10]:= iad["DegreesOfFreedom"]

Out[10]= 0

In[11]:= iad["NonIdentifiableParameters"]

Out[11]= {}

In[12]:= endTime = AbsoluteTime[]
         N[endTime - startTime]

Out[12]= 3.6879518837211211 × 10^9

Out[13]= 0.685957
```
