# Supplementary material for: GenSSI 2.0: multi-experiment structural identifiability analysis of SBML models
Source: Bioinformatics. 2017 Nov 30;34(8):1421–3. doi: 10.1093/bioinformatics/btx735 (PMC5905618; doi:10.1093/bioinformatics/btx735)
Supplement: Supplementary Data [file btx735_suppl_data.zip › EAR.zip!/EAR/GoodwinExtended.pdf]

In[1]:=
```
Needs["IdentifiabilityAnalysis`"]
startTime = AbsoluteTime[]
```

Out[2]= $3.6879579109293680\times 10^9$

In[3]:=
```
vars = {x1, x2, x3, x4}
```

Out[3]= {x1, x2, x3, x4}

In[4]:=
```
params = {p1, p2, p3, p4, p5, p6, p7, p8, p9}
```

Out[4]= {p1, p2, p3, p4, p5, p6, p7, p8, p9}

In[5]:=
```
sys = {x1'[t] == -p4 * x1[t] + p1 / (p2 + x4[t]),
  x2'[t] == p5 * x1[t] - p6 * x2[t],
  x3'[t] == p7 * x2[t] - p8 * x3[t],
  x4'[t] == p3 (p7 x2[t] - p8 x3[t]) x4[t] / x3[t],
  x1[0] == 0.3, x2[0] == 0.9, x3[0] == 1.3, x4[0] == p9}
```

Out[5]= $\left\{x1'[t] == -p4\,x1[t] + \frac{p1}{p2 + x4[t]},\ x2'[t] == p5\,x1[t] - p6\,x2[t],\ x3'[t] == p7\,x2[t] - p8\,x3[t],\ x4'[t] == \frac{p3\,(p7\,x2[t] - p8\,x3[t])\,x4[t]}{x3[t]},\ x1[0] == 0.3,\ x2[0] == 0.9,\ x3[0] == 1.3,\ x4[0] == p9\right\}$

The original system can be rewritten by introducing an additional state variable $z(t) = x_3(t)^{p_3}$ which implies the relations $z'(t) = p_3\, z(t)/x_3(t)\, x_3'(t) = p_3\, z(t)/x_3(t)\,(p_7\, x_2(t) - p_8\, x_3(t))$ and $z(0) = x_3(0)^{p_3} = 1.3^{p_3}$. The last expression is further rewritten as $z(0) = q$, where q is a new parameter. Hence,

Now, from an input-output perspective the extended system in sys is equivalent to the original one except for the relation $1.3^{p_3} = q$, which is an invertible mapping, i.e., there is a one-one relation correspondence between p_3 and q.

The extended list of state variables

The extended list of parameters

and the Identifiability Analysis of the extended system (except the non-rational relation $1.3^{p_3} = q$)

In[6]:=
```
iad = IdentifiabilityAnalysis[{sys, output}, vars, params, t]
```

Out[6]= IdentifiabilityAnalysisData[False, <>]

The analyzed system is non-identifiable

In[7]:=
```
iad["IdentifiableQ"]
```

Out[7]= False

with one degree of freedom, i.e., by fixing one of the non-identified parameters to a numerical value the non-identifiability will be resolved.

In[8]:=
```
iad["DegreesOfFreedom"]
```

Out[8]= 9

The non-identifiable parameters are

In[9]:=
```
iad["NonIdentifiableParameters"]
```

Out[9]=
```
{p1, p2, p3, p4, p5, p6, p7, p8, p9}
```

We note that p3 is not among these, which means that by using the non-included relation $1.3^{p_3} = q$ the value of q can be fixed and the original (and equivalent extended system) can be rendered identifiable.

In[10]:=
```
endTime = AbsoluteTime[]
```

Out[10]= $3.6879579114615571 \times 10^9$

In[11]:=
```
N[endTime - startTime]
```

Out[11]=
```
0.532189
```
