# Supplementary material for: GenSSI 2.0: multi-experiment structural identifiability analysis of SBML models
Source: Bioinformatics. 2017 Nov 30;34(8):1421–3. doi: 10.1093/bioinformatics/btx735 (PMC5905618; doi:10.1093/bioinformatics/btx735)
Supplement: Supplementary Data [file btx735_suppl_data.zip › EAR.zip!/EAR/HighDimNonLin.pdf]

```
In[1]:= Needs["IdentifiabilityAnalysis`"]
        startTime = AbsoluteTime[]

Out[2]= 3.6879585282619276 × 10^9

In[3]:= vars = {x1, x2, x3, x4, x5, x6, x7, x8, x9,
          x10, x11, x12, x13, x14, x15, x16, x17, x18, x19, x20}

Out[3]= {x1, x2, x3, x4, x5, x6, x7, x8, x9, x10,
         x11, x12, x13, x14, x15, x16, x17, x18, x19, x20}

In[4]:= params = {vm, km, p1, p2, p3, p4, p5, p6, p7, p8, p9, p10, p11, p12, p13,
          p14, p15, p16, p17, p18, p19, p20, x01, x02, x03, x04, x05, x06, x07, x08,
          x09, x010, x011, x012, x013, x014, x015, x016, x017, x018, x019, x020}

Out[4]= {vm, km, p1, p2, p3, p4, p5, p6, p7, p8, p9, p10, p11, p12, p13, p14,
         p15, p16, p17, p18, p19, p20, x01, x02, x03, x04, x05, x06, x07, x08,
         x09, x010, x011, x012, x013, x014, x015, x016, x017, x018, x019, x020}
```

```
In[5]:= sys = {x1'[t] == -vm * x1[t] / (km + x1[t]) - p1 * x1[t],
    x2'[t] == p1 * x1[t] - p2 * x2[t],
    x3'[t] == p2 * x2[t] - p3 * x3[t],
    x4'[t] == p3 * x3[t] - p4 * x4[t],
    x5'[t] == p4 * x4[t] - p5 * x5[t],
    x6'[t] == p5 * x5[t] - p6 * x6[t],
    x7'[t] == p6 * x6[t] - p7 * x7[t],
    x8'[t] == p7 * x7[t] - p8 * x8[t],
    x9'[t] == p8 * x8[t] - p9 * x9[t],
    x10'[t] == p9 * x9[t] - p10 * x10[t],
    x11'[t] == p10 * x10[t] - p11 * x11[t],
    x12'[t] == p11 * x11[t] - p12 * x12[t],
    x13'[t] == p12 * x12[t] - p13 * x13[t],
    x14'[t] == p13 * x13[t] - p14 * x14[t],
    x15'[t] == p14 * x14[t] - p15 * x15[t],
    x16'[t] == p15 * x15[t] - p16 * x16[t],
    x17'[t] == p16 * x16[t] - p17 * x17[t],
    x18'[t] == p17 * x17[t] - p18 * x18[t],
    x19'[t] == p18 * x18[t] - p19 * x19[t],
    x20'[t] == p19 * x19[t] - p20 * x20[t],
    x1[0] == x01, x2[0] == x02, x3[0] == x03, x4[0] == x04, x5[0] == x05,
    x6[0] == x06, x7[0] == x07, x8[0] == x08, x9[0] == x09, x10[0] == x010,
    x11[0] == x011, x12[0] == x012, x13[0] == x013, x14[0] == x014, x15[0] == x015,
    x16[0] == x016, x17[0] == x017, x18[0] == x018, x19[0] == x019, x20[0] == x020}
```

Out[5]= $\{x1'[t] == -p1\, x1[t] - \frac{vm\, x1[t]}{km + x1[t]}, x2'[t] == p1\, x2[t] - p2\, x2[t],$

```
x3'[t] == p2 x2[t] - p3 x3[t], x4'[t] == p3 x3[t] - p4 x4[t], x5'[t] == p4 x4[t] - p5 x5[t],
x6'[t] == p5 x5[t] - p6 x6[t], x7'[t] == p6 x6[t] - p7 x7[t], x8'[t] == p7 x7[t] - p8 x8[t],
x9'[t] == p8 x8[t] - p9 x9[t], x10'[t] == -p10 x10[t] + p9 x9[t],
x11'[t] == p10 x10[t] - p11 x11[t], x12'[t] == p11 x11[t] - p12 x12[t],
x13'[t] == p12 x12[t] - p13 x13[t], x14'[t] == p13 x13[t] - p14 x14[t],
x15'[t] == p14 x14[t] - p15 x15[t], x16'[t] == p15 x15[t] - p16 x16[t],
x17'[t] == p16 x16[t] - p17 x17[t], x18'[t] == p17 x17[t] - p18 x18[t],
x19'[t] == p18 x18[t] - p19 x19[t], x20'[t] == p19 x19[t] - p20 x20[t],
x1[0] == x01, x2[0] == x02, x3[0] == x03, x4[0] == x04, x5[0] == x05,
x6[0] == x06, x7[0] == x07, x8[0] == x08, x9[0] == x09, x10[0] == x010,
x11[0] == x011, x12[0] == x012, x13[0] == x013, x14[0] == x014, x15[0] == x015,
x16[0] == x016, x17[0] == x017, x18[0] == x018, x19[0] == x019, x20[0] == x020}
```

```
In[6]:= output = {x1[t], x2[t], x3[t], x4[t], x5[t], x6[t], x7[t], x8[t], x9[t], x10[t],
    x11[t], x12[t], x13[t], x14[t], x15[t], x16[t], x17[t], x18[t], x19[t], x20[t]}

Out[6]= {x1[t], x2[t], x3[t], x4[t], x5[t], x6[t], x7[t], x8[t], x9[t], x10[t], x11[t],
  x12[t], x13[t], x14[t], x15[t], x16[t], x17[t], x18[t], x19[t], x20[t]}

In[7]:= iad = IdentifiabilityAnalysis[{sys, output}, vars, params, t,]

Out[7]= IdentifiabilityAnalysisData[True, <>]

In[8]:= iad["IdentifiableQ"]

Out[8]= True
```

In[9]:=
```
iad["DegreesOfFreedom"]
```
Out[9]= 0

In[10]:=
```
iad["NonIdentifiableParameters"]
```
Out[10]= { }

In[11]:=
```
endTime = AbsoluteTime[]
N[endTime - startTime]
```
Out[11]= $3.6879585305617043 \times 10^{9}$

Out[12]= 2.29978

In[13]:=
