# Supplementary material for: GenSSI 2.0: multi-experiment structural identifiability analysis of SBML models
Source: Bioinformatics. 2017 Nov 30;34(8):1421–3. doi: 10.1093/bioinformatics/btx735 (PMC5905618; doi:10.1093/bioinformatics/btx735)
Supplement: Supplementary Data [file btx735_suppl_data.zip › EAR.zip!/EAR/HIV.pdf]

```
In[194]:= Needs["IdentifiabilityAnalysis`"]
          startTime = AbsoluteTime[]

Out[195]= 3.6878012376241517 × 10^9

In[196]:= vars = {x1, x2, x3, x4}

Out[196]= {x1, x2, x3, x4}

In[197]:= params = {b, c, d, q1, q2, k1, k2, w1, w2}

Out[197]= {b, c, d, q1, q2, k1, k2, w1, w2}

In[198]:= sys = {x1'[t] == -b * x1[t] * x4[t] - d * x1[t] + u1[t],
            x2'[t] == b * q1 * x1[t] * x4[t] - k1 * x2[t] - w1 * x2[t],
            x3'[t] == b * q2 * x1[t] * x4[t] + k1 * x2[t] - w2 * x3[t],
            x4'[t] == -c * x4[t] + k2 * x3[t],
            x1[0] == 0, x2[0] == 0, x3[0] == 0, x4[0] == 0}

Out[198]= {x1′[t] == u1[t] - d x1[t] - b x1[t] x4[t],
           x2′[t] == -k1 x2[t] - w1 x2[t] + b q1 x1[t] x4[t],
           x3′[t] == k1 x2[t] - w2 x3[t] + b q2 x1[t] x4[t],
           x4′[t] == k2 x3[t] - c x4[t], x1[0] == 0, x2[0] == 0, x3[0] == 0, x4[0] == 0}

In[199]:= output = {x1[t], x2[t]}

Out[199]= {x1[t], x2[t]}

In[200]:= iad = IdentifiabilityAnalysis[{sys, output}, vars, params, t, {u1}]

Out[200]= IdentifiabilityAnalysisData[False, <>]

In[201]:= iad["IdentifiableQ"]

Out[201]= False

In[202]:= iad["DegreesOfFreedom"]

Out[202]= 8

In[203]:= iad["NonIdentifiableParameters"]

Out[203]= {b, c, k1, k2, q1, q2, w1, w2}

In[204]:= endTime = AbsoluteTime[]
          N[endTime - startTime]

Out[204]= 3.6878012378959397 × 10^9

Out[205]= 0.271788
```
