# Supplementary material for: GenSSI 2.0: multi-experiment structural identifiability analysis of SBML models
Source: Bioinformatics. 2017 Nov 30;34(8):1421–3. doi: 10.1093/bioinformatics/btx735 (PMC5905618; doi:10.1093/bioinformatics/btx735)
Supplement: Supplementary Data [file btx735_suppl_data.zip › EAR.zip!/EAR/JAK-STAT.pdf]

In[13]:=
```
Needs["IdentifiabilityAnalysis`"]
startTime = AbsoluteTime[]
```

Out[14]= $3.6883641001975584 \times 10^9$

In[15]:=
```
vars = {x1, x2, x3, x4, x5, x6, x7, x8, x9}
```

Out[15]=
```
{x1, x2, x3, x4, x5, x6, x7, x8, x9}
```

In[16]:=
```
params = {p1, p2, p3, p4, p5, p6, p7}
```

Out[16]=
```
{p1, p2, p3, p4, p5, p6, p7}
```

In[17]:=
```
sys = {x1'[t] == (p7 * p4 * x9[t]) / p6 - p1 * x1[t] * u1[t],
   x2'[t] == -2 * p2 * x2[t]^2 + p1 * x1[t] * u2[t],
   x3'[t] == p2 * x2[t]^2 - p3 * x3[t],
   x4'[t] == -(p7 * p4 * x4[t] - p6 * p3 * x3[t]) / p7,
   x5'[t] == -p4 * (x5[t] - 2 * x4[t]),
   x6'[t] == p4 * (x5[t] - x6[t]),
   x7'[t] == p4 * (x6[t] - x7[t]),
   x8'[t] == p4 * (x7[t] - x8[t]),
   x9'[t] == p4 * (x8[t] - x9[t]),
   x1[0] == p5, x2[0] == 0, x3[0] == 0, x4[0] == 0,
   x5[0] == 0, x6[0] == 0, x7[0] == 0, x8[0] == 0, x9[0] == 0}
```

Out[17]=

$$\Big\{x1'[t] == -p1\, u1[t]\, x1[t] + \frac{p4\, p7\, x9[t]}{p6},\ x2'[t] == p1\, u2[t]\, x1[t] - 2\, p2\, x2[t]^2,$$
$$x3'[t] == p2\, x2[t]^2 - p3\, x3[t],\ x4'[t] == \frac{p3\, p6\, x3[t] - p4\, p7\, x4[t]}{p7},$$
$$x5'[t] == -p4\, (-2\, x4[t] + x5[t]),\ x6'[t] == p4\, (x5[t] - x6[t]),$$
$$x7'[t] == p4\, (x6[t] - x7[t]),\ x8'[t] == p4\, (x7[t] - x8[t]),$$
$$x9'[t] == p4\, (x8[t] - x9[t]),\ x1[0] == p5,\ x2[0] == 0,\ x3[0] == 0,$$
$$x4[0] == 0,\ x5[0] == 0,\ x6[0] == 0,\ x7[0] == 0,\ x8[0] == 0,\ x9[0] == 0\Big\}$$

In[18]:=
```
output = {(x2[t] + 2 * x3[t]) / p5, (x1[t] + x2[t] + 2 * x3[t]) / p5;}
```

Out[18]=

$$\left\{\frac{x2[t] + 2\, x3[t]}{p5},\ Null\right\}$$

In[19]:=
```
iad = IdentifiabilityAnalysis[{sys, output}, vars, params, t, {u1, u2}]
```

Out[19]=
```
IdentifiabilityAnalysisData[False, <>]
```

In[20]:=
```
iad["IdentifiableQ"]
```

Out[20]=
```
False
```

In[21]:=
```
iad["DegreesOfFreedom"]
```

Out[21]=
```
2
```

In[22]:=
```
iad["NonIdentifiableParameters"]
```

Out[22]=
```
{p6, p7}
```

In[23]:=
```
endTime = AbsoluteTime[]
N[endTime - startTime]
```

Out[23]= $3.6883641014999065\times10^{9}$

Out[24]= 1.30235
