# Supplementary material for: GenSSI 2.0: multi-experiment structural identifiability analysis of SBML models
Source: Bioinformatics. 2017 Nov 30;34(8):1421–3. doi: 10.1093/bioinformatics/btx735 (PMC5905618; doi:10.1093/bioinformatics/btx735)
Supplement: Supplementary Data [file btx735_suppl_data.zip › EAR.zip!/EAR/NFkB.pdf]

```
In[1]:= Needs["IdentifiabilityAnalysis`"]
        startTime = AbsoluteTime[]

Out[2]= 3.6883703117513825 × 10^9

In[3]:= vars = {x1, x2, x3, x4, x5, x6, x7, x8, x9, x10, x11, x12, x13, x14, x15}

Out[3]= {x1, x2, x3, x4, x5, x6, x7, x8, x9, x10, x11, x12, x13, x14, x15}

In[4]:= params = {a1, a2, a3, t1, t2, c1a, c2a, c3a, c4a, c5a, c6a, c1,
          c2, c3, c4, c5, k3, kprod, kdeg, kv, i1, e2a, i1a, e1a, c1c, c2c,
          c3c, x01, x06, x07, x08, x09, x010, x011, x012, x014, x015, NF}

Out[4]= {a1, a2, a3, t1, t2, c1a, c2a, c3a, c4a, c5a, c6a, c1,
         c2, c3, c4, c5, k3, kprod, kdeg, kv, i1, e2a, i1a, e1a, c1c, c2c,
         c3c, x01, x06, x07, x08, x09, x010, x011, x012, x014, x015, NF}

In[5]:= sys = {x1'[t] == kprod - kdeg * x1[t] - k1 * x1[t] * u1[t],
        x2'[t] == -k3 * x2[t] - kdeg * x2[t] - a2 * x2[t] * x10[t] + t1 * x4[t] -
            a3 * x2[t] * x13[t] + t2 * x5[t] + (k1 * x1[t] - k2 * x2[t] * x8[t]) * u2[t],
        x3'[t] == k3 * x2[t] - kdeg * x3[t] + k2 * x2[t] * x8[t] * u3[t],
        x4'[t] == a2 * x2[t] * x10[t] - t1 * x4[t],
        x5'[t] == a3 * x2[t] * x13[t] - t2 * x5[t],
        x6'[t] == c6a * x13[t] - a1 * x6[t] * x10[t] + t2 * x5[t] - i1 * x6[t],
        x7'[t] == i1 * kv * x6[t] - a1 * x11[t] * x7[t],
        x8'[t] == c4 * x9[t] - c5 * x8[t],
        x9'[t] == c2 + c1 * x7[t] - c3 * x9[t],
        x10'[t] == -a2 * x2[t] * x10[t] - a1 * x10[t] * x6[t] +
            c4a * x12[t] - c5a * x10[t] - i1a * x10[t] + e1a * x11[t],
        x11'[t] == -a1 * x11[t] * x7[t] + i1a * kv * x10[t] - e1a * kv * x11[t],
        x12'[t] == c2a + c1a * x7[t] - c3a * x12[t],
        x13'[t] == a1 * x10[t] * x6[t] - c6a * x13[t] - a3 * x2[t] * x13[t] + e2a * x14[t],
        x14'[t] == a1 * x11[t] * x7[t] - e2a * kv * x14[t],
        x15'[t] == c2c + c1c * x7[t] - c3c * x15[t],
          x1[0] == x01, x2[0] == 0, x3[0] == 0, x4[0] == 0, x5[0] == 0, x6[0] == x06,
          x7[0] == x07, x8[0] == x08, x9[0] == x09, x10[0] == x010, x11[0] == x011,
          x12[0] == x012, x13[0] == NF, x14[0] == x014, x15[0] == x015}

Out[5]= {x1′[t] == kprod - kdeg x1[t] - k1 u1[t] x1[t],
         x2′[t] == -k3 x2[t] - kdeg x2[t] - a2 x10[t] x2[t] - a3 x13[t] x2[t] +
           t1 x4[t] + t2 x5[t] + u2[t] (k1 x1[t] - k2 x2[t] x8[t]),
         x3′[t] == k3 x2[t] - kdeg x3[t] + k2 u3[t] x2[t] x8[t],
         x4′[t] == a2 x10[t] x2[t] - t1 x4[t], x5′[t] == a3 x13[t] x2[t] - t2 x5[t],
         x6′[t] == c6a x13[t] + t2 x5[t] - i1 x6[t] - a1 x10[t] x6[t],
         x7′[t] == i1 kv x6[t] - a1 x11[t] x7[t], x8′[t] == -c5 x8[t] + c4 x9[t],
         x9′[t] == c2 + c1 x7[t] - c3 x9[t], x10′[t] ==
          -c5a x10[t] - i1a x10[t] + e1a x11[t] + c4a x12[t] - a2 x10[t] x2[t] - a1 x10[t] x6[t],
         x11′[t] == i1a kv x10[t] - e1a kv x11[t] - a1 x11[t] x7[t],
         x12′[t] == c2a - c3a x12[t] + c1a x7[t],
         x13′[t] == -c6a x13[t] + e2a x14[t] - a3 x13[t] x2[t] + a1 x10[t] x6[t],
         x14′[t] == -e2a kv x14[t] + a1 x11[t] x7[t], x15′[t] == c2c - c3c x15[t] + c1c x7[t],
         x1[0] == x01, x2[0] == 0, x3[0] == 0, x4[0] == 0, x5[0] == 0, x6[0] == x06,
         x7[0] == x07, x8[0] == x08, x9[0] == x09, x10[0] == x010, x11[0] == x011,
         x12[0] == x012, x13[0] == NF, x14[0] == x014, x15[0] == x015}
```

```
In[6]:= iad = IdentifiabilityAnalysis[{sys, output}, vars, params, t, {u1, u2, u3}]
Out[6]= $Aborted

iad["IdentifiableQ"]
False

iad["DegreesOfFreedom"]
38

iad["NonIdentifiableParameters"]
{a1, a2, a3, c1, c1a, c1c, c2, c2a, c2c, c3, c3a, c3c, c4,
 c4a, c5, c5a, c6a, e1a, e2a, i1, i1a, k3, kdeg, kprod, kv, NF,
 t1, t2, x01, x010, x011, x012, x014, x015, x06, x07, x08, x09}

endTime = AbsoluteTime[]
N[endTime - startTime]
```

$3.6879594152006310 \times 10^{9}$

```
17.9392
```
