# Supplementary material for: GenSSI 2.0: multi-experiment structural identifiability analysis of SBML models
Source: Bioinformatics. 2017 Nov 30;34(8):1421–3. doi: 10.1093/bioinformatics/btx735 (PMC5905618; doi:10.1093/bioinformatics/btx735)
Supplement: Supplementary Data [file btx735_suppl_data.zip › EAR.zip!/EAR/NGF-Erk-extended.pdf]

```
In[14]:= Needs["IdentifiabilityAnalysis`"]
         startTime = AbsoluteTime[]
```

Out[15]= $3.6891568006721086 \times 10^9$

```
In[16]:= vars = {x1, x2, x3, x4, x5, x6}

Out[16]= {x1, x2, x3, x4, x5, x6}

In[17]:= params = {k1, k2, k4, k5, k7, k9, k11, k3TrkA0,
           k6Ras0, k8Raf0, k10Mek0, sErk0, sK, n, NGF0, p1, p2}

Out[17]= {k1, k2, k4, k5, k7, k9, k11, k3TrkA0,
          k6Ras0, k8Raf0, k10Mek0, sErk0, sK, n, NGF0, p1, p2}
```

In[18]:=
```
sys = {x1'[t] == -x1[t] * k2 - NGF0 * k1 * (x1[t] - k3TrkA0),
  x2'[t] == -x2[t] * k5 - (x2[t] - k6Ras0) * (k4 + (x1[t] * p1) / (x6[t] + p1)),
  x3'[t] == x2[t] * (k8Raf0 - x3[t]) - k7 * x3[t],
  x4'[t] == x3[t] * (k10Mek0 - x4[t]) - k9 * x4[t],
  x5'[t] == -k11 * x5[t] - x4[t] * (x5[t] - sErk0),
   x6'[t] == (n * x6[t] / x5[t]) * (-k11 * x5[t] - x4[t] * (x5[t] - sErk0)),
  x1[0] == 0,
  x2[0] == (k4 * k6Ras0) / (k4 + k5),
  x3[0] == (k4 * k6Ras0 * k8Raf0) / ((k4 + k5) * (k7 + (k4 * k6Ras0) / (k4 + k5))),
  x4[0] == (k4 * k6Ras0 * k8Raf0 * k10Mek0) / ((k4 + k5) * (k7 + (k4 * k6Ras0) / (k4 + k5)) *
       (k9 + (k4 * k6Ras0 * k8Raf0) / ((k4 + k5) * (k7 + (k4 * k6Ras0) / (k4 + k5))))),
  x5[0] == (k4 * k6Ras0 * k8Raf0 * k10Mek0 * sErk0) /
     ((k4 + k5) * (k7 + (k4 * k6Ras0) / (k4 + k5)) * (k11 + (k4 * k6Ras0 * k8Raf0 * k10Mek0) /
          ((k4 + k5) * (k7 + (k4 * k6Ras0) / (k4 + k5)) * (k9 +
               (k4 * k6Ras0 * k8Raf0) / ((k4 + k5) * (k7 + (k4 * k6Ras0) / (k4 + k5)))))) *
       (k9 + (k4 * k6Ras0 * k8Raf0) / ((k4 + k5) * (k7 + (k4 * k6Ras0) / (k4 + k5))))),
   x6[
    0] ==
   p2}
```

Out[18]=

$$\Big\{x1'[t] == -k2\, x1[t] - k1\, NGF0\, (-k3TrkA0 + x1[t]),$$

$$x2'[t] == -k5\, x2[t] - (-k6Ras0 + x2[t]) \left(k4 + \frac{p1\, x1[t]}{p1 + x6[t]}\right),$$

$$x3'[t] == x2[t]\, (k8Raf0 - x3[t]) - k7\, x3[t],\ x4'[t] == x3[t]\, (k10Mek0 - x4[t]) - k9\, x4[t],$$

$$x5'[t] == -k11\, x5[t] - x4[t]\, (-sErk0 + x5[t]),$$

$$x6'[t] == \frac{n\, (-k11\, x5[t] - x4[t]\, (-sErk0 + x5[t]))\, x6[t]}{x5[t]},$$

$$x1[0] == 0,\ x2[0] == \frac{k4\, k6Ras0}{k4 + k5},\ x3[0] == \frac{k4\, k6Ras0\, k8Raf0}{(k4 + k5)\left(\frac{k4\, k6Ras0}{k4+k5} + k7\right)},$$

$$x4[0] == \frac{k10Mek0\, k4\, k6Ras0\, k8Raf0}{(k4 + k5)\left(\frac{k4\, k6Ras0}{k4+k5} + k7\right)\left(\frac{k4\, k6Ras0\, k8Raf0}{(k4+k5)\left(\frac{k4\, k6Ras0}{k4+k5}+k7\right)} + k9\right)},\ x5[0] ==$$

$$\frac{k10Mek0\, k4\, k6Ras0\, k8Raf0\, sErk0}{(k4 + k5)\left(\frac{k4\, k6Ras0}{k4+k5} + k7\right)\left(\frac{k4\, k6Ras0\, k8Raf0}{(k4+k5)\left(\frac{k4\, k6Ras0}{k4+k5}+k7\right)} + k9\right)\left(k11 + \frac{k10Mek0\, k4\, k6Ras0\, k8Raf0}{(k4+k5)\left(\frac{k4\, k6Ras0}{k4+k5}+k7\right)\left(\frac{k4\, k6Ras0\, k8Raf0}{(k4+k5)\left(\frac{k4\, k6Ras0}{k4+k5}+k7\right)}+k9\right)}\right)}$$

$$,\ x6[0] == p2\Big\}$$

In[19]:=
```
output = {x5[t]}
```

Out[19]= {x5[t]}

In[20]:=
```
iad = IdentifiabilityAnalysis[{sys, output}, vars, params, t]
```

Out[20]= IdentifiabilityAnalysisData[False, <>]

In[21]:=
```
iad["IdentifiableQ"]
```

Out[21]= False

In[22]:=
```
iad["DegreesOfFreedom"]
```
Out[22]=
```
4
```

In[23]:=
```
iad["NonIdentifiableParameters"]
```
Out[23]=
```
{k1, k2, k3TrkA0, NGF0, p1, p2, sK}
```

In[24]:=
```
endTime = AbsoluteTime[]
N[endTime - startTime]
```
Out[24]= $3.6891568030911393 \times 10^{9}$

Out[25]=
```
2.41903
```

In[26]:=
