# Supplementary material for: GenSSI 2.0: multi-experiment structural identifiability analysis of SBML models
Source: Bioinformatics. 2017 Nov 30;34(8):1421–3. doi: 10.1093/bioinformatics/btx735 (PMC5905618; doi:10.1093/bioinformatics/btx735)
Supplement: Supplementary Data [file btx735_suppl_data.zip › EAR.zip!/EAR/Pharmacokinetics.pdf]

```
In[1]:= Needs["IdentifiabilityAnalysis`"]
        startTime = AbsoluteTime[]

Out[2]= 3.6884675522843210 × 10^9

In[3]:= vars = {x1, x2, x3, x4}

Out[3]= {x1, x2, x3, x4}

In[4]:= params = {a1, a2, b1, b2, ka, kc, vm, c0, g}

Out[4]= {a1, a2, b1, b2, ka, kc, vm, c0, g}

In[5]:= sys = {x1 '[t] == a1 * (x2[t] - x1[t]) - ka * vm * x1[t] / (kc * ka + kc * x3[t] + ka * x1[t]),
           x2 '[t] == a2 * (x1[t] - x2[t]),
           x3 '[t] == b1 * (x4[t] - x3[t]) - kc * vm * x3[t] / (kc * ka + kc * x3[t] + ka * x1[t]),
           x4 '[t] == b2 * (x3[t] - x4[t]),
           x1[0] == c0, x2[0] == 0, x3[0] == g * c0, x4[0] == 0}
```

Out[5]= $\left\{x1'[t] == a1\,(-x1[t] + x2[t]) - \frac{ka\ vm\ x1[t]}{ka\ kc + ka\ x1[t] + kc\ x3[t]},\ x2'[t] == a2\,(x1[t] - x2[t]),\right.$

$x3'[t] == -\frac{kc\ vm\ x3[t]}{ka\ kc + ka\ x1[t] + kc\ x3[t]} + b1\,(-x3[t] + x4[t]),$

$\left. x4'[t] == b2\,(x3[t] - x4[t]),\ x1[0] == c0,\ x2[0] == 0,\ x3[0] == c0\ g,\ x4[0] == 0\right\}$

```
In[6]:= output = {x1[t], x4[t]}

Out[6]= {x1[t], x4[t]}

In[7]:= iad = IdentifiabilityAnalysis[{sys, output}, vars, params, t]

Out[7]= IdentifiabilityAnalysisData[True, <>]

In[8]:= iad["IdentifiableQ"]

Out[8]= True

In[9]:= iad["DegreesOfFreedom"]

Out[9]= 0

In[10]:= iad["NonIdentifiableParameters"]

Out[10]= {}

In[11]:= endTime = AbsoluteTime[]
         N[endTime - startTime]

Out[11]= 3.6884675529534635 × 10^9

Out[12]= 0.669143

In[13]:=
```
