# Supplementary material for: GenSSI 2.0: multi-experiment structural identifiability analysis of SBML models
Source: Bioinformatics. 2017 Nov 30;34(8):1421–3. doi: 10.1093/bioinformatics/btx735 (PMC5905618; doi:10.1093/bioinformatics/btx735)
Supplement: Supplementary Data [file btx735_suppl_data.zip › EAR.zip!/EAR/PharmacokineticsPoly.pdf]

```
In[14]:= Needs["IdentifiabilityAnalysis`"]
         startTime = AbsoluteTime[]

Out[15]= 3.6878020383067895 × 10^9

In[16]:= vars = {x1, x2, x3, x4, x5}

Out[16]= {x1, x2, x3, x4, x5}

In[17]:= params = {a1, a2, b1, b2, ka, kc, vm, c0, g}

Out[17]= {a1, a2, b1, b2, ka, kc, vm, c0, g}

In[18]:= sys = {x1'[t] == -a1 * (x1[t] - x2[t]) - ka * vm * x1[t] * x5[t],
            x2'[t] == a2 * (x1[t] - x2[t]),
            x3'[t] == -b1 * (x3[t] - x4[t]) - kc * vm * x3[t] * x5[t],
            x4'[t] == b2 * (x3[t] - x4[t]),
            x5'[t] == ka * x5[t] ^2 * (a1 * (x1[t] - x2[t]) + ka * vm * x1[t] * x5[t]) +
              kc * x5[t] ^2 * (b1 * (x3[t] - x4[t]) + kc * vm * x3[t] * x5[t]),
            x1[0] == c0, x2[0] == 0, x3[0] == g * c0, x4[0] == 0,
            x5[0] == 1 / (c0 * ka + ka * kc + c0 * g * kc)}

Out[18]= {x1′[t] == -a1 (x1[t] - x2[t]) - ka vm x1[t] x5[t], x2′[t] == a2 (x1[t] - x2[t]),
          x3′[t] == -b1 (x3[t] - x4[t]) - kc vm x3[t] x5[t], x4′[t] == b2 (x3[t] - x4[t]),
          x5′[t] == ka x5[t]^2 (a1 (x1[t] - x2[t]) + ka vm x1[t] x5[t]) +
            kc x5[t]^2 (b1 (x3[t] - x4[t]) + kc vm x3[t] x5[t]), x1[0] == c0,
          x2[0] == 0, x3[0] == c0 g, x4[0] == 0, x5[0] == 1/(c0 ka + c0 g kc + ka kc)}

In[19]:= output = {x1[t], x4[t]}

Out[19]= {x1[t], x4[t]}

In[20]:= iad = IdentifiabilityAnalysis[{sys, output}, vars, params, t, {u1, u2}]

Out[20]= IdentifiabilityAnalysisData[True, <>]

In[21]:= iad["IdentifiableQ"]

Out[21]= True

In[22]:= iad["DegreesOfFreedom"]

Out[22]= 0

In[23]:= iad["NonIdentifiableParameters"]

Out[23]= {}

In[24]:= endTime = AbsoluteTime[]
         N[endTime - startTime]

Out[24]= 3.6878020392079253 × 10^9

Out[25]= 0.901136

In[26]:=
```
