# Supplementary material for: GenSSI 2.0: multi-experiment structural identifiability analysis of SBML models
Source: Bioinformatics. 2017 Nov 30;34(8):1421–3. doi: 10.1093/bioinformatics/btx735 (PMC5905618; doi:10.1093/bioinformatics/btx735)
Supplement: Supplementary Data [file btx735_suppl_data.zip › EAR.zip!/EAR/Thyroid1.pdf]

```
In[1]:= Needs["IdentifiabilityAnalysis`"]
        startTime = AbsoluteTime[]

Out[2]= 3.6879595833189266 × 10^9

In[3]:= vars = {x1, x2, x3}

Out[3]= {x1, x2, x3}

In[4]:= params = {k02, k03, k12, k13, k21, k31, V1}

Out[4]= {k02, k03, k12, k13, k21, k31, V1}

In[5]:= sys = {x1 '[t] == k13 * x3[t] + k12 * x2[t] - (k21 + k31) * x1[t] + u1[t],
           x2 '[t] == k21 * x1[t] - (k12 + k02) * x2[t],
           x3 '[t] == k31 * x1[t] - (k13 + k03) * x3[t],
           x1[0] == 0, x2[0] == 0, x3[0] == 0}

Out[5]= {x1′[t] == u1[t] - (k21 + k31) x1[t] + k12 x2[t] + k13 x3[t],
         x2′[t] == k21 x1[t] - (k02 + k12) x2[t],
         x3′[t] == k31 x1[t] - (k03 + k13) x3[t], x1[0] == 0, x2[0] == 0, x3[0] == 0}

In[6]:= output = {x1[t] / V1}

Out[6]= {x1[t]/V1}

In[7]:= iad = IdentifiabilityAnalysis[{sys, output}, vars, params, t, {u1}]

Out[7]= IdentifiabilityAnalysisData[False, <>]

In[8]:= iad["IdentifiableQ"]

Out[8]= False

In[9]:= iad["DegreesOfFreedom"]

Out[9]= 1

In[10]:= iad["NonIdentifiableParameters"]

Out[10]= {k02, k03, k12, k13, k21, k31}

In[11]:= endTime = AbsoluteTime[]
         N[endTime - startTime]

Out[11]= 3.6879595836883030 × 10^9

Out[12]= 0.369376

In[13]:=
```
