# Supplementary material for: GenSSI 2.0: multi-experiment structural identifiability analysis of SBML models
Source: Bioinformatics. 2017 Nov 30;34(8):1421–3. doi: 10.1093/bioinformatics/btx735 (PMC5905618; doi:10.1093/bioinformatics/btx735)
Supplement: Supplementary Data [file btx735_suppl_data.zip › EAR.zip!/EAR/Thyroid2.pdf]

```
In[66]:= Needs["IdentifiabilityAnalysis`"]
         startTime = AbsoluteTime[]

Out[67]= 3.6878026538464756 × 10^9
```

```
In[68]:= vars = {x1, x2, x3}

Out[68]= {x1, x2, x3}
```

```
In[69]:= params = {k02, k03, k12, k13, k21, k31, V1}

Out[69]= {k02, k03, k12, k13, k21, k31, V1}
```

```
In[70]:= sys = {x1 '[t] == k13 * x3[t] + k12 * x2[t] - (k21 + k31) * x1[t] + u1[t],
            x2 '[t] == k21 * x1[t] - (k12 + k02) * x2[t],
            x3 '[t] == k31 * x1[t] - (k13 + k03) * x3[t],
            x1[0] == 0, x2[0] == 0, x3[0] == 0}

Out[70]= {x1′[t] == u1[t] - (k21 + k31) x1[t] + k12 x2[t] + k13 x3[t],
          x2′[t] == k21 x1[t] - (k02 + k12) x2[t],
          x3′[t] == k31 x1[t] - (k03 + k13) x3[t], x1[0] == 0, x2[0] == 0, x3[0] == 0}
```

```
In[71]:= {x1′[t] == u1[t] - (k21 + k31) x1[t] + k12 x2[t] + k13 x3[t],
          x2′[t] == k21 x1[t] - (k02 + k12) x2[t], x3′[t] == k31 x1[t] - (k03 + k13) x3[t],
          x1[0] == 0, x2[0] == 0, x3[0] == c0 g, x4[0] == 0, x5[0] == 1/(c0 ka + c0 g kc + ka kc)}

Out[71]= {x1′[t] == u1[t] - (k21 + k31) x1[t] + k12 x2[t] + k13 x3[t],
          x2′[t] == k21 x1[t] - (k02 + k12) x2[t], x3′[t] == k31 x1[t] - (k03 + k13) x3[t],
          x1[0] == 0, x2[0] == 0, x3[0] == c0 g, x4[0] == 0, x5[0] == 1/(c0 ka + c0 g kc + ka kc)}
```

```
In[72]:= output = {x1[t] / V1}

Out[72]= {x1[t]/V1}
```

```
In[73]:= iad = IdentifiabilityAnalysis[{sys, output}, vars, params, t, {u1}]

Out[73]= IdentifiabilityAnalysisData[False, <>]
```

```
In[74]:= iad["IdentifiableQ"]

Out[74]= False
```

```
In[75]:= iad["DegreesOfFreedom"]

Out[75]= 1
```

```
In[76]:= iad["NonIdentifiableParameters"]

Out[76]= {k02, k03, k12, k13, k21, k31}
```

```
In[77]:= endTime = AbsoluteTime[]
         N[endTime - startTime]

Out[77]= 3.6878026542596730 × 10^9

Out[78]= 0.413197
```

```
In[79]:=
```
