# Supplementary material for: GenSSI 2.0: multi-experiment structural identifiability analysis of SBML models
Source: Bioinformatics. 2017 Nov 30;34(8):1421–3. doi: 10.1093/bioinformatics/btx735 (PMC5905618; doi:10.1093/bioinformatics/btx735)
Supplement: Supplementary Data [file btx735_suppl_data.zip › EAR.zip!/EAR/Transfection_2State.pdf]

```
In[14]:= Needs["IdentifiabilityAnalysis`"]
         startTime = AbsoluteTime[]

Out[15]= 3.6888122180721281 × 10^9

In[16]:= vars = {x1, x2}

Out[16]= {x1, x2}

In[17]:= params = {d, kTL, b, m0}

Out[17]= {d, kTL, b, m0}

In[18]:= sys = {x1'[t] == -d * x1[t],
           x2'[t] == kTL * x1[t] - b * x2[t],
           x1[0] == m0, x2[0] == 0}

Out[18]= {x1′[t] == -d x1[t], x2′[t] == kTL x1[t] - b x2[t], x1[0] == m0, x2[0] == 0}

In[19]:= output = {x2[t]}

Out[19]= {x2[t]}

In[20]:= iad = IdentifiabilityAnalysis[{sys, output}, vars, params, t]

Out[20]= IdentifiabilityAnalysisData[False, <>]

In[21]:= iad["IdentifiableQ"]

Out[21]= False

In[22]:= iad["DegreesOfFreedom"]

Out[22]= 1

In[23]:= iad["NonIdentifiableParameters"]

Out[23]= {kTL, m0}

In[24]:= endTime = AbsoluteTime[]
         N[endTime - startTime]

Out[24]= 3.6888122182335621 × 10^9

Out[25]= 0.161434

In[26]:=
```
