# Supplementary material for: GenSSI 2.0: multi-experiment structural identifiability analysis of SBML models
Source: Bioinformatics. 2017 Nov 30;34(8):1421–3. doi: 10.1093/bioinformatics/btx735 (PMC5905618; doi:10.1093/bioinformatics/btx735)
Supplement: Supplementary Data [file btx735_suppl_data.zip › EAR.zip!/EAR/Transfection_2State_MultiExp.pdf]

```
In[1]:= Needs["IdentifiabilityAnalysis`"]
        startTime = AbsoluteTime[]

Out[2]= 3.7143131216768538 × 10^9

In[3]:= vars = {x1, x2, x3, x4, x5, x6, x7, x8}

Out[3]= {x1, x2, x3, x4, x5, x6, x7, x8}

In[4]:= params = {d, b, kTL, m0, m1, g1, m2, g2, m3, g3, m4, g4}

Out[4]= {d, b, kTL, m0, m1, g1, m2, g2, m3, g3, m4, g4}

In[5]:= sys = {x1'[t] == -d * x1[t],
          x2'[t] == kTL * x1[t] - b * x2[t],
          x3'[t] == -d * x3[t],
          x4'[t] == kTL * x3[t] - b * x4[t],
          x5'[t] == -d * x5[t],
          x6'[t] == kTL * x5[t] / 2 - b * x6[t],
          x7'[t] == -d * x7[t],
          x8'[t] == kTL * x7[t] - 3 * b * x8[t] / 4,
          x1[0] == m1, x2[0] == 0, x3[0] == m1 / 2,
          x4[0] == 0, x5[0] == m1, x6[0] == 0, x7[0] == m1, x8[0] == 0}
```

Out[5]= $\{x1'[t] == -d\, x1[t],\ x2'[t] == kTL\, x1[t] - b\, x2[t],\ x3'[t] == -d\, x3[t],$
$x4'[t] == kTL\, x3[t] - b\, x4[t],\ x5'[t] == -d\, x5[t],\ x6'[t] == \frac{1}{2} kTL\, x5[t] - b\, x6[t],$
$x7'[t] == -d\, x7[t],\ x8'[t] == kTL\, x7[t] - \frac{3}{4} b\, x8[t],\ x1[0] == m1,\ x2[0] == 0,$
$x3[0] == \frac{m1}{2},\ x4[0] == 0,\ x5[0] == m1,\ x6[0] == 0,\ x7[0] == m1,\ x8[0] == 0\}$

```
In[6]:= output = {x2[t]}

Out[6]= {x2[t]}

In[7]:= iad = IdentifiabilityAnalysis[{sys, output}, vars, params, t]

Out[7]= IdentifiabilityAnalysisData[False, <>]

In[8]:= iad["IdentifiableQ"]

Out[8]= False

In[9]:= iad["DegreesOfFreedom"]

Out[9]= 9

In[10]:= iad["NonIdentifiableParameters"]

Out[10]= {g1, g2, g3, g4, kTL, m0, m1, m2, m3, m4}
```

In[11]:=
```
endTime = AbsoluteTime[]
N[endTime - startTime]
```

Out[11]= $3.7143131224302231\times 10^{9}$

Out[12]= 0.753369

In[13]:=
