# Supplementary material for: GenSSI 2.0: multi-experiment structural identifiability analysis of SBML models
Source: Bioinformatics. 2017 Nov 30;34(8):1421–3. doi: 10.1093/bioinformatics/btx735 (PMC5905618; doi:10.1093/bioinformatics/btx735)
Supplement: Supplementary Data [file btx735_suppl_data.zip › EAR.zip!/EAR/Transfection_4State.pdf]

```
In[55]:= Needs["IdentifiabilityAnalysis`"]
         startTime = AbsoluteTime[]

Out[56]= 3.7143127095913888 × 10^9

In[57]:= vars = {x1, x2, x3, x4}

Out[57]= {x1, x2, x3, x4}

In[58]:= params = {d1, d2, d3, b, kTL, m0, e0}

Out[58]= {d1, d2, d3, b, kTL, m0, e0}

In[59]:= sys = {x1'[t] == -d * x1[t] - d2 * x1[t] * x3[t],
           x2'[t] == kTL * x1[t] - b * x2[t],
           x3'[t] == d3 * x4[t] - d2 * x1[t] * x3[t],
           x4'[t] == -d3 * x4[t] + d2 * x1[t] * x3[t],
           x1[0] == m0, x2[0] == 0, x3[0] == e0, x4[0] == 0}

Out[59]= {x1′[t] == -d x1[t] - d2 x1[t] x3[t], x2′[t] == kTL x1[t] - b x2[t],
          x3′[t] == -d2 x1[t] x3[t] + d3 x4[t], x4′[t] == d2 x1[t] x3[t] - d3 x4[t],
          x1[0] == m0, x2[0] == 0, x3[0] == e0, x4[0] == 0}

In[60]:= output = {x2[t]}

Out[60]= {x2[t]}
```

```
In[61]:= iad = IdentifiabilityAnalysis[{sys, output}, vars, params, t]
```

```
MatrixRank::nmod :
  {{0, 0, 0, 0, 0, 0, 0, 0, 1, 0, 0}, {0, 0, 0, 0, 1358158970, 0, 0, 311676514 + 1835807115 d, 1610432727, 0, 0}} is not valid
      modulo 2147483629. ≫

MatrixRank::nmod :
  {{0, 0, 0, 0, 0, 0, 0, 0, 1, 0, 0}, {0, 0, 0, 0, 1358158970, 0, 0, 311676514 + 1524130601 d, 1610432727, 0, 0}, {0, 1204721082, 0,
        972145393, ≪3≫, 432407064 + 1594346015 d + 623353028 d^2, 588508773, 340106561, 0}, {0, 627551479 +
          1885525094 d, 0, 1189840539 + 1175338236 d, 55067104 + 616024789 d + 1358158970 d^2, 0, 0, 183081669 +
          1866091235 d + 1801547468 d^2 + 1524130601 d^3, 2046111463, 1348922464 + 1467270507 d, 313795259}}
      is not valid modulo 2147483629. ≫

MatrixRank::nmod :
  {{0, 0, 0, 0, 0, 0, 0, 0, 1, 0, 0}, {0, 0, 0, 0, 1358158970, 0, 0, 311676514 + 1212454087 d, 1610432727, 0, 0}, ≪5≫, {0, 738162205 +
          2084211904 d + 1700161549 d^2 + 412472409 d^3 + 661565600 d^4 + 1361608024 d^5, 1205026758 + 1819024125 d +
          642240198 d^2 + 1722830683 d^3, ≪5≫, 1899826236, 1734191209 + 511316814 d + 1023578827 d^2 + 67706457 d^3 +
          1577884849 d^4 + 106844263 d^5, 812111100 + 2049016504 d + 507625799 d^2 + 1107172809 d^3 + 411961627 d^4}}
      is not valid modulo 2147483629. ≫

General::stop : Further output of MatrixRank::nmod will be suppressed during this calculation. ≫

RowReduce::nmod :
  {{0, 0, 0, 0, 0, 0, 0, 0, 1, 0, 0}, ≪9≫, {0, 1216356917 + 1461167345 d + 598684078 d^2 + 1031139942 d^3 + 895937528 d^4 +
          166983657 d^5 + 1706372156 d^6 + 993175182 d^7 + 105071593 d^8, ≪7≫, 2087300481 + 1738388257 d + 503746821
            d^2 + 1365168199 d^3 + 1412293484 d^4 + 617290169 d^5 + 812499857 d^6 + 426859651 d^7 + 913475420 d^8,
        1423785579 + 1759607543 d + 786258779 d^2 + 964408865 d^3 + 1318244575 d^4 + 1475521986 d^5 + 805072898 d^6 +
          1588272450 d^7}} is not valid modulo 2147483629. ≫

Transpose::nmtx : The first two levels of the one-dimensional list
      {NullSpace[RowReduce[{{0, 0, 0, 0, 0, 0, 0, 0, 1, 0, 0}, {0, 0, 0, 0, 1358158970, 311676514 + 900777573 d, 0, 311676514 +
              900777573 d, 1610432727, 0, 0}, ≪7≫, {0, ≪1≫, ≪7≫, ≪1≫, 1896029611 + 1998813256 d + ≪3≫ +
              802961958 Power[≪2≫] + 196332736 Power[≪2≫]}, {0, ≪9≫, 1423785579 + 1759607543 d + 786258779
                Power[≪2≫] + 964408865 Power[≪2≫] + 1318244575 Power[≪2≫] + 1475521986 Power[≪2≫] +
              805072898 Power[≪2≫] + 1588272450 Power[≪2≫]}}, ≪1≫], ≪1≫]}
      cannot be transposed. ≫

Part::take : Cannot take positions 1 through 7 in
      NullSpace[{{0, 0, 0, 0, 0, -1, 0, 1, 0, 0, 0}, {0, 0, 0, 0, 0, 0, 0, 0, 1, 0, 0}, {0, 0, 0, 0, 0, 0, -1, 0, 0, 1, 0}, {0, 0, 0, 0, 0, 0, 0, 0, 0, 0, 1}}.
            Transpose[{NullSpace[RowReduce[{≪11≫}, Rule[≪2≫]], Modulus → 2147483629]}], Modulus → 2147483629].
        NullSpace[RowReduce[{{0, 0, 0, 0, 0, 0, 0, 0, 1, 0, 0}, ≪10≫}, Modulus → 2147483629], Modulus → 2147483629].
      ≫
```

```
Out[61]= IdentifiabilityAnalysisData[False, <>]

In[62]:= iad["IdentifiableQ"]

Out[62]= False

In[63]:= iad["DegreesOfFreedom"]

Out[63]= 2
```

In[64]:=
```
iad["NonIdentifiableParameters"]
```
Out[64]= {b, d1, d2, d3, e0, kTL, m0}

In[65]:=
```
endTime = AbsoluteTime[]
N[endTime - startTime]
```
Out[65]= $3.7143127117249377 \times 10^{9}$

Out[66]= 2.13355

In[67]:=
