# Supplementary material for: GenSSI 2.0: multi-experiment structural identifiability analysis of SBML models
Source: Bioinformatics. 2017 Nov 30;34(8):1421–3. doi: 10.1093/bioinformatics/btx735 (PMC5905618; doi:10.1093/bioinformatics/btx735)
Supplement: Supplementary Data [file btx735_suppl_data.zip › EAR.zip!/EAR/Transfection_4State_Transformation.pdf]

```
In[1]:= Needs["IdentifiabilityAnalysis`"]
        startTime = AbsoluteTime[]

Out[2]= 3.7143134082115011 × 10^9

In[3]:= vars = {x1, x2, x3}

Out[3]= {x1, x2, x3}

In[4]:= params = {d, d2tm0, d3, b, kTLtm0, e0dm0}

Out[4]= {d, d2tm0, d3, b, kTLtm0, e0dm0}

In[5]:= sys = {x1'[t] == -d1 * x1[t] - d2m0 * x1[t] * x3[t],
          x2'[t] == kTLtm0 * x1[t] - b * x2[t],
          x3'[t] == d3 * e0dm0 - d3 * x3[t] - d2tm0 * x1[t] * x3[t],
          x1[0] == 1, x2[0] == 0, x3[0] == e0dm0}

Out[5]= {x1′[t] == -d1 x1[t] - d2m0 x1[t] x3[t], x2′[t] == kTLtm0 x1[t] - b x2[t],
         x3′[t] == d3 e0dm0 - d3 x3[t] - d2tm0 x1[t] x3[t], x1[0] == 1, x2[0] == 0, x3[0] == e0dm0}

In[6]:= output = {x2[t]}

Out[6]= {x2[t]}
```

In[7]:=
```
iad = IdentifiabilityAnalysis[{sys, output}, vars, params, t]
```

```
MatrixRank::nmod :
  {{0, 0, 0, 0, 2147483628 d1, 0, 0, 1, 0}, {0, 0, 0, 0, 1 + 441510061 d1, 0, 2021483916 + 125999713 d1, 1705973568, 125999713 d2m0}
      } is not valid modulo 2147483629. ≫

MatrixRank::nmod : ≪1≫ is not valid modulo 2147483629. ≫

MatrixRank::nmod :
  {{0, 0, 0, 0, 2147483627 d1, 0, 0, 1, 0}, ≪6≫, {0, ≪7≫, 2086513770 d2m0 + 177833867 d1 d2m0 + 958205955 d1^2 d2m0 +
      825027329 d1^3 d2m0 + 2065771611 d1^4 d2m0 + 753532917 d1^5 d2m0 + 906167840 d1^6 d2m0 + 36087405 d2m0^2 +
      2141434200 d1 d2m0^2 + ≪10≫ + 253383446 d1 d2m0^4 + 190947060 d1^2 d2m0^4 + 56057245 d1^3 d2m0^4 +
      343984856 d2m0^5 + 940352233 d1 d2m0^5 + 1575751637 d1^2 d2m0^5 + 1839182757 d2m0^6 + 345370266 d1 d2m0^6 +
      151650050 d2m0^7}} is not valid modulo 2147483629. ≫

General::stop : Further output of MatrixRank::nmod will be suppressed during this calculation. ≫

RowReduce::nmod : {{0, 0, 0, 0, 2147483627 d1, 0, 0, 1, 0}, ≪8≫} is not valid modulo 2147483629. ≫

Transpose::nmtx : The first two levels of the one-dimensional list
    {NullSpace[RowReduce[{{0, 0, 0, 0, 2147483627 d1, 0, 0, 1, 0}, {0, ≪8≫}, ≪5≫, {0, ≪7≫, ≪1≫}, {0, ≪7≫, 1287297962
          d2m0 + 801289929 d1 d2m0 + 1966717609 Power[≪2≫] d2m0 + 627822680 Power[≪2≫] d2m0 +
        1066637382 Power[≪2≫] d2m0 + 653590196 Power[≪2≫] d2m0 + ≪24≫ + 1916936665 Power[≪2≫] +
        701360135 d1 Power[≪2≫] + 1884760917 Power[≪2≫] Power[≪2≫] + 1445383961 Power[≪2≫] +
        1436460448 d1 Power[≪2≫] + 1571862631 Power[≪2≫]}}, ≪1≫], ≪1≫]}
    cannot be transposed. ≫

Part::take : Cannot take positions 1 through 6 in
    NullSpace[{{0, 0, 0, 0, 0, 0, 1, 0, 0}, {0, 0, 0, 0, 0, 0, 0, 1, 0}, {0, 0, 0, 0, 0, -1, 0, 0, 1}}.Transpose[{NullSpace[RowReduce[{≪9≫},
          Rule[≪2≫]], Modulus → 2147483629]}], Modulus → 2147483629].NullSpace[RowReduce[{{0, 0, 0, 0,
      2147483627 d1, 0, 0, 1, 0}, ≪7≫, {0, ≪1≫, ≪5≫, 35612030, 1287297962 d2m0 + 801289929 d1 d2m0 +
        1966717609 Power[≪2≫] d2m0 + ≪30≫ + 1445383961 Power[≪2≫] + 1436460448 d1 Power[≪2≫] +
        1571862631 Power[≪2≫]}}, ≪1≫], ≪1≫]. ≫
```

Out[7]=
```
IdentifiabilityAnalysisData[False, <>]
```

In[8]:=
```
iad["IdentifiableQ"]
```

Out[8]=
```
False
```

In[9]:=
```
iad["DegreesOfFreedom"]
```

Out[9]=
```
2
```

In[10]:=
```
iad["NonIdentifiableParameters"]
```

Out[10]=
```
{b, d, d2tm0, d3, e0dm0, kTLtm0}
```

In[11]:=
```
endTime = AbsoluteTime[]
N[endTime - startTime]
```

Out[11]=
```
3.7143134168499305 × 10^9
```

Out[12]=
```
8.63843
```

In[13]:=
