# Supplementary material for: GenSSI 2.0: multi-experiment structural identifiability analysis of SBML models
Source: Bioinformatics. 2017 Nov 30;34(8):1421–3. doi: 10.1093/bioinformatics/btx735 (PMC5905618; doi:10.1093/bioinformatics/btx735)
Supplement: Supplementary Data [file btx735_suppl_data.zip › GenSSI-code.zip!/GenSSI-code/GenSSI-refman.pdf]

GenSSI

0

Generated by Doxygen 1.8.13

Contents

1 GenSSI 2.0 General Documentation 2

1.1 Introduction 2

1.2 Availability 2

1.3 Installation 3

2 Model Definition & Simulation 4

2.1 Model Definition 4

2.2 Model Analysis 5

2.3 Conversion Utilities 6

3 Code Organization 13

3.1 Directory Structure 13

3.2 Document Creation 13

4 Hierarchical Index 14

4.1 Class Hierarchy 14

5 Class Index 14

5.1 Class List 14

6 Class Documentation 14

6.1 SBMLode Class Reference 14

7 File Documentation **23**

7.1 Auxiliary/amiciStructToSource.m File Reference 23
7.2 Auxiliary/genssiAskForConfirmation.m File Reference 24
7.3 Auxiliary/genssiCheckExpCondition.m File Reference 25
7.4 Auxiliary/genssiCheckModel.m File Reference 26
7.5 Auxiliary/genssiComputeLieDerivatives.m File Reference 27
7.6 Auxiliary/genssiComputeReducedTableau.m File Reference 29
7.7 Auxiliary/genssiComputeTableau.m File Reference 30
7.8 Auxiliary/genssiGetCandForTransformation.m File Reference 31
7.9 Auxiliary/genssiGetSymChar.m File Reference 32
7.10 Auxiliary/genssiOrderTableau.m File Reference 33
7.11 Auxiliary/genssiPolySys.m File Reference 35
7.12 Auxiliary/genssiRemoveZeroColumns.m File Reference 36
7.13 Auxiliary/genssiRemoveZeroElementsC.m File Reference 37
7.14 Auxiliary/genssiRemoveZeroElementsR.m File Reference 38
7.15 Auxiliary/genssiRemoveZeroRows.m File Reference 39
7.16 Auxiliary/genssiReportInputs.m File Reference 40
7.17 Auxiliary/genssiReportResults.m File Reference 41
7.18 Auxiliary/genssiStructToSource.m File Reference 42
7.19 Auxiliary/genssiTableauImage.m File Reference 43
7.20 Auxiliary/genssiTransposeModel.m File Reference 44
7.21 genssiMain.m File Reference 45
7.22 genssiMultiExperiment.m File Reference 46
7.23 genssiStartup.m File Reference 47
7.24 genssiToPolynomial.m File Reference 47
7.25 genssiTransformation.m File Reference 48
7.26 genssiUserSpecificDefaults.m File Reference 50
7.27 SBMLimporter/computeBracketLevel.m File Reference 50

Index **53**

1 GenSSI 2.0 General Documentation

1.1 Introduction

GenSSI is a Matlab implementation of generating series for structural identifiability as defined in

- Chiş, O.-T., Banga, J.R. and Balsa-Canto, E. (2011) Structural Identifiability of Systems Biology Models: A Critical Comparison of Methods, PLoS ONE, 6, e27755.
- Chiş, O., Banga, J.R. and Balsa-Canto, E. (2011) GenSSI: a software toolbox for structural identifiability analysis of biological models, Bioinformatics, 27, 2610-2611.

With GenSSI, the user can specify differential equation models in terms of symbolic variables in Matlab and then analyze the models to determine which parameters are globally or locally identifiable. In addition, there are some utilities for importing models from SBML, or converting models to polynomial form and for creating multi-experiment models.

1.2 Availability

The sources for GenSSI are accessible as

- Source `tarball`
- Source `zipball`
- Git repository on `github`

Once you've obtained your copy check out the Installation

1.2.1 Obtaining GenSSI via the Git versioning system

In order to always stay up to date with the latest GenSSI versions, simply pull it from our Git repository and recompile it when a new release is available. For more information about Git checkout their `website`

The Git repository can currently be found at `https://github.com/genssi-developer/GenSSI` and a direct clone is possible via

```
git clone https://github.com/genssi-developer/GenSSI.git GenSSI
```

1.2.2 License Conditions

This software is available under the `BSD license`

Copyright (c) 2016, Oana-Teodora Chiş, Julio R. Banga, Eva Balsa-Canto, Thomas S. Ligon, Fabian Fröhlich and Jan Hasenauer. All rights reserved.

Redistribution and use in source and binary forms, with or without modification, are permitted provided that the following conditions are met:

- Redistributions of source code must retain the above copyright notice, this list of conditions and the following disclaimer.
- Redistributions in binary form must reproduce the above copyright notice, this list of conditions and the following disclaimer in the documentation and/or other materials provided with the distribution.

THIS SOFTWARE IS PROVIDED BY THE COPYRIGHT HOLDERS AND CONTRIBUTORS "AS IS" AND ANY EXPRESS OR IMPLIED WARRANTIES, INCLUDING, BUT NOT LIMITED TO, THE IMPLIED WARRANTIES OF MERCHANTABILITY AND FITNESS FOR A PARTICULAR PURPOSE ARE DISCLAIMED. IN NO EVENT SHALL THE COPYRIGHT HOLDER OR CONTRIBUTORS BE LIABLE FOR ANY DIRECT, INDIRECT, INCIDENTAL, SPECIAL, EXEMPLARY, OR CONSEQUENTIAL DAMAGES (INCLUDING, BUT NOT LIMITED TO, PROCUREMENT OF SUBSTITUTE GOODS OR SERVICES; LOSS OF USE, DATA, OR PROFITS; OR BUSINESS INTERRUPTION) HOWEVER CAUSED AND ON ANY THEORY OF LIABILITY, WHETHER IN CONTRACT, STRICT LIABILITY, OR TORT (INCLUDING NEGLIGENCE OR OTHERWISE) ARISING IN ANY WAY OUT OF THE USE OF THIS SOFTWARE, EVEN IF ADVISED OF THE POSSIBILITY OF SUCH DAMAGE.

1.3 Installation

If GenSSI was downloaded as a zip, it needs to be unpacked in a convenient directory. If GenSSI was obtained via cloning of the git repository, no further unpacking is necessary.

Models are generally stored in

```
GenSSI/Examples
```

but GenSSI should be able to find them in any directory that is is the Matlab path.

When a model is analyzed, GenSSI stores the results in

```
GenSSI/Examples
```

To use GenSSI, start Matlab and add the GenSSI direcory to the Matlab path. To add all toolbox directories to the Matlab path, execute the Matlab script

```
genssiStartup.m
```

To use the SBML import, libSBML (`http://sbml.org/Software/libSBML/Downloading_libSBML#MATLAB`) has to be downloaded and the directory has to be included in the MATLAB path. To store the installation for further Matlab sessions, the path can be saved via

```
savepath
```

2 Model Definition & Simulation

In the following we will give a detailed overview how to specify models in GenSSI and how to call the code for analyzing the model. We use the Goodwin oscillator as an example.

2.1 Model Definition

This manual will guide the user to specify models in MATLAB. For example implementations, see the models in the example directory.

2.1.1 Header

The model definition needs to be defined as a function which returns a struct with all symbolic definitions and options.

```
function [model] = Goodwin()
```

2.1.2 States

Create the respective symbolic variables. The name of the symbolic variable can be chosen arbitrarily.

```
syms x1 x2 x3
```

Create the state vector containing all states:

```
model.sym.x = [x1;x2;x3];
```

2.1.3 Parameters

Create the respective symbolic variables. The name of the symbolic variable can be chosen arbitrarily.

```
syms p1 p2 p3 p4 p5 p6 p7 p8
```

Create the parameter vector:

```
model.sym.p = [p1;p2;p3;p4;p5;p6;p7;p8]
```

2.1.4 Equations

Define the equations of the model.

```
model.sym.xdot=[-p4*x1+p1/(p2+x3^p3);...
                p5*x1-p6*x2;,...
                p7*x2-p8*x3];
```

2.1.5 Controls

Define the controls.

```
model.sym.g=[];
```

Note that the matrix of controls can by empty ([]) or contain one or more controls. Each row must have the same length as the number of states (model.sym.x), and there must be one row for each control.

2.1.6 Observables

Define the observables.

```
model.sym.y = [x1;x2;x3];
```

2.1.7 Initial Conditions

Define the initial conditions.

```
model.sym.x0 = [0.3;0.9;1.3];
```

2.2 Model Analysis

The model can then be analyzed by calling genssiMain. The first parameter is the name of the model, the second parameter (Nder) is the number of derivatives to be calculated, and the third parameter (Par) is the list of parameters to be considered for identifiability. If the second parameter is absent a default number of derivatives is used. If the third parameter is absent, the full list of parameters will be used.

The fourth parameter is a struct containing options for the analysis run. If absent, defaults will be used. The options are:

```
options.verbose (default=true); maximum (verbose) information in results file
options.noRank (default=false); no rank calculation (for speed with loss)
options.closeFigure (default=false); closes figures after storing it
options.store (default=false); stores the results of analysis in a file
options.reportCompTime (default=false); reports the computation time
```

When GenSSI is run for the first time, and genssiStartup is run, a file named

```
genssiUserSpecificDefaults.m
```

If you have specific default options that you would like to reuse, you can either change the defaults in this file, or supply options to genssiMain.

```
genssiMain('modelName',Nder,Par,options);
```

The function genssiMain will call the model function, which puts the model struct in memory. After that, it will call all other GenSSI functions required to annalyze the model.

To make this easier to find, we have also provided a simple "run" script, "runGoodwin".

```
% runGoodwin runs the structural identifiability analysis for the
% model of the Goodwin oscillator as introduced by
%
%    Goodwin, B.C. (1965). Oscillatory behavior in enzymatic control
%              processes, Adv. Enzyme Regul. (3), 425-428.
% Confirm execution
genssiAskForConfirmation(2);
% Structural identifiability analysis (for a subset of the parameters)
genssiMain('Goodwin',5);
```

In addition to this, in order to avoid switching to the GenSSI/Examples/Goodwin directory, you can also call runExample from the main directory.

```
runExample('Goodwin');
```

2.3 Conversion Utilities

The GenSSI package also includes some functions for converting models from one format to another.

2.3.1 Convert from SBML Format

```
ODE = SBMLode(modelName);
ODE.writeAMICI(modelName);
```

SBMLode and writeAMICI convert an SBML model to the common GenSSI/AMICI format.

modelName: name of the SBML and GenSSI model (string).

Reminder: To use the SBML import, libSBML (http://sbml.org/Software/libSBML/Downloading_libSBML#MATLA[illegible] has to be downloaded and the directory has to be included in the MATLAB path.

The input SBML model should be stored in the directory

```
GenSSI/Examples
```

Note: GenSSI now uses AMICI format. This reduces duplication and gives us full functionality of the AMICI routines. Because of this, the number of derivatives and the parameters considered for identifiability have been moved from the model definition to the call to genssiMain. These parameters are more a matter of analysis than of model definition.

Note: There are limitations to this conversion. The SBML model contains a list of all parameters used by the model, but GenSSI needs a list of parameters to be considered for analysis. It may be necessary to manually edit the GenSSI model after conversion. Both GenSSI and AMICI require specification of the observables, but this is not defined in SBML. If SBMLode finds rules that are not used in the model, it will assume that they were intended as observables. In any case, it is advisable to inspect the observables and modify them as needed.

As an example for this conversion, we have chosen to use an SMBL model from the biomodels database. We begin by accessing the web site http://www.ebi.ac.uk/biomodels-main/ searching for MAPK and dowloading the file BIOMD0000000010.xml. Our SBML model is now contained in

```
BIOMD0000000010.xml
```

To convert this SBML model to a GenSSI model with the same name, we execute the script

```
runBIOMD0000000010;
```

The script file includes several default values. Here, the full list of parameters is contained in ODE.parameter (later model.sym.p) is copied to Par (parameter in genssiMain) and reduced in size to the list of parameters Par for which identifiability analysis is performed. Using all 22 parameters is in principle possible, but does not work on a desktop computer with 16GB RAM, because the size of the Jacobian grows exponentially with number of of parameters. In addition to the parameters, we set the observables (ODE.observable and model.sym.y = [MAPK, MAPK_P, and MAPK_PP]), and the number of Lie derivatives (parameter Nder = 6 in genssiMain).

```
% runBIOMD0000000010 runs the structural identifiability analysis for the
% model of the MAP kinase cascade described in
%
%    Kholodenko BN (2000). Negative feedback and ultrasensitivity can
%    bring about oscillations in the mitogen-activated protein kinase
%    cascades. Eur. J. Biochem., 267(6): 1583-1588.
%
% The model has been downloaded from the BioModels Database
% (http://biomodels.caltech.edu/BIOMD0000000010) as an SBML file. This SBML
% file is converted in the GenSSI format before analysis using the libSBML
% see (http://sbml.org/Software/libSBML/Downloading_libSBML). This file is
% extended by observables and renamed.
% Model name
modelName = 'BIOMD0000000010';
% Import of SBML model
ODE = SBMLode([modelName '.xml']);
% Definition of observables
ODE.observable = ODE.state([2,5,8]);
% Writing of model to AMICI format
% (which is consistent with GenSSI)
ODE.writeAMICI(modelName);
% Rename file
movefile([modelName '_syms.m'],[modelName '.m']);
% Confirm execution
genssiAskForConfirmation(600);
% Structural identifiability analysis (for a subset of the model parameters)
genssiMain(modelName,6,ODE.parameter([1,5,11,13,19,21]));
```

This model can now be analyzed using the fucntionality of GenSSI:

```
genssiMain(modelName,6,ODE.parameter([1,5,11,13,19,21]));
```

The results show that 3 of the 6 parameters are locally identifiable and the other 3 are globally identifiable.

2.3.2 Convert to Polynomial Format

```
genssiToPolynomial(modelNameIn,modelNameOut)
```

genssiToPolynomial converts a model, expressed in terms of rational expressions, to pure polynomial format. This increases the number of state variables, but can sometimes significantly reduce the computational overhead for analyzing the model.

modeNameIn: name of model to be converted (string).

modelNameOut: name of model to be created (string).

An example of conversion to polynomial format and analysis is contained in

```
runArabidopsisPoly.m
```

```
% runArabidopsisPoly runs the structural identifiability analysis for the
% model of the circadian clock in Arabidopsis Thaliana as introduced by
%
%    Locke et al. (2005). Modeling genetic networks with noisy and
%    varied experimental data: the circadian clock in Arabidopsis
%    thaliana. Journal of Theoretical Biology 234: 383-393.
%
% To simplify the symbolic calculations, the model is converted to
% polynomial form, meaning that the vector field of the autonomous
% dynamics (f) and the control vector (g) are polynomial. To achieve this,
% the dimensionality of the model is increased.
% Copy Arabidopsis.m to this folder
copyfile(fullfile('..','Arabidopsis','Arabidopsis.m'),'Arabidopsis.m');
% Transform model to polynomial
genssiToPolynomial('Arabidopsis','ArabidopsisPoly');
% Confirm execution
genssiAskForConfirmation(80);
% Symbolic parameters for identifiability analysis
syms p1 p2 p5 p8 p10 p11 p12 p15 p18 p19 p26 p27
% Structural identifiability analysis (for a subset of the parameters)
genssiMain('ArabidopsisPoly',7,[p1;p2;p5;p8;p10;p11;p12;p15;p18;p26;p27]);
```

2.3.3 Create Multi-Experiment Model

```
genssiMultiExperiment(modelNameIn,mExDef,modelNameOut)
```

genssiMultiExperiment converts a GenSSI model to a new GenSSI model based on a multi-experiment definition.

modelNameIn: the name of the input model (a string).

mExDef: the name of a multi-experiment definition file (string).

modelNameOut: the name of the output model (a string).

In chemistry, it is often possible for the chemist to arbitrarily change certain parameters, such as temperature, pressure, and the concentration of specific substances. For example, in a continuous-flow stirred tank reactor (CFSTR), the feed flow provides a constant feed of substances, at a rate that can be chosen as needed. This situation has led to the concept of "controls", which are also used in identifiability analysis. From the point of theory, the controls are variables that can be changed at will, so they have a very strong positive influence on the identifiability of a model.

In contrast, in biology, certain parameters and feed rates may be varied, but most often not arbitrarily. Often, these parameters can be varied discretely by creating a new experiment with new substances or substance concentrations. Now, in identifiability, we would like to analyze multiple experiments in one model. The result is a model for which the identifiability lies somewhere between the models without controls and those with controls.

Now we will explain the parameters used by the multi-experiment model creation on the basis of a specific example.

We begin with a model for mRNA, including translation and degradation. The model definition is:

```
 function model = Transfection_2State()
     % Transfection_2State provides the GenSSI implementation of the model
     % for mRNA transfection introduced by
     %
     %    Leonhardt et al. (2013). Single-cell mRNA transfection
     %    studies: delivery, kinetics and statistics by numbers,
     %    Nanomedicine: Nanotechnology, Biology and Medicine. 10(4):679-88.
     %
     % The ODE is given by
     %
     %    d[mRNA|/dt = -d*[mRNA],                         [mRNA](0) = mRNA0
     %    d[GFP]/dt  = kTL*[M]*[uSyn] - b*[GFP]*[uDeg],   [GFP](0)  = 0
     %
     % in which [mRNA] and [GFP] denote the concentrations of GFP-mRNA and
     % GFP-protein, respectively. The controls are the concentation of a
     % protein synthesis inhibitor, [uSyn], and the concentation of a
     % protein degradation inhibitor, [uDeg].
     % Symbolic variables
     syms mRNA GFP
     syms d b kTL mRNA0
     % Parameters
     model.sym.p = [d;b;kTL;mRNA0];
     % State variables
     model.sym.x = [mRNA;GFP];
     % Control vectors (g)
     %                 uSyn    uDeg
     model.sym.g = [       0,      0   % mRNA
                    kTL*mRNA,-b*GFP]; % GFP
     % Autonomous dynamics (f)
     model.sym.xdot = [-d*mRNA;0];
     % Initial conditions
     model.sym.x0 = [mRNA0;0];
     % Observables
     model.sym.y = [GFP];
end
```

The states (model.sym.x) are mRNA and GFP (green fluorescent protein), of which only GFP is observed (model.sym.y). The differential equations (model.sym.xdot) define degradation of mRNA (-d∗mRNA), degradation of GFP (-b∗GFP) and translation of mRNA to GFP (kTL∗mRNA). Note that translation and GFP degradation have been defined as controls (model.sym.g).

Based on this model, we define a total of 4 experiments, in the experiment definition function. The expriments are defined by modifying the controls and the initial conditions. The expriments are:

1) original configuration

2) change in transfection (mRNA0) via x0: The initial concentration of mRNA is changed.

3) change in translation via control u1 = uInh: The rate of translation is changed, for example by treating the cell with an antibiotic.

4) change in GFP degradation via control u2 = uDeg: The rate of GFP degradation i changed, for example by using destabilized GFP (d2eGFP) instead of normal GFP (eGFP).

The experiment definition is:

```
function expCond = ExperimentalConditions_2State()
    % ExperimentalConditions_2State defines four different experimental
    % conditions for the model for mRNA transfection introduced by
    %
    %    Leonhardt et al. (2013). Single-cell mRNA transfection
    %    studies: delivery, kinetics and statistics by numbers,
    %    Nanomedicine: Nanotechnology, Biology and Medicine. 10(4):679-88.
    %
    % Experimental conditions
    % (1) transfection without perturbations
    % (2) reduction of amount of transfection (mRNA0)
    % (3) reduction of translation (uSyn)
    % (4) reduction of protein degration (uDeg)
    % Symbolic variables
    syms mRNA0
    % Input values
    %     Condition  (1)   (2)   (3)   (4)
    expCond.sym.u = [1.0, 1.0, 0.5, 1.0    % uSyn
                     1.0, 1.0, 1.0, .75]; % uDeg
    % Initial conditions
    %     Condition      (1)       (2)       (3)     (4)
    expCond.sym.x0 = [ mRNA0, 0.5*mRNA0, mRNA0, mRNA0    % [mRNA](0)
                          0,          0,      0,      0]; % [GFP](0)
end
```

The number of expriments is coded in multiExp.Nexp=4.

The variable multiExp.sym.u defines the changes in the controls and the variable multiExp.sym.x0 defines the changes in the initial conditions. Both of these variables contain one row for each experiment and one column for each state variable. In the first experiment (original configuration), the controls are 1 and the initial mRNA concentration is mRNA0. In the second expriment (change in transfection), the initial concentration of mRNA is changed. In the third experiment (change in translation), the rate of translation is changed by changing the value of the first control. In the fourth experiment (change in GFP degradation), the rate of GFP degradation is changed by changing the value of the second control.

The original model is converted to the multi-experiment model by means of this line of code:

```
runTransfection_2State_MultiExp.m
```

and

```
genssiMultiExperiment('Transfection_2State','ExperimentalConditions_2State','
      Transfection_2State_MultiExp');
```

```
% runExperimentalConditions_2State performs the structural identifiability
% analysis for the model for mRNA transfection introduced by
%
%    Leonhardt et al. (2013). Single-cell mRNA transfection
%    studies: delivery, kinetics and statistics by numbers,
%    Nanomedicine: Nanotechnology, Biology and Medicine. 10(4):679-88.
%
% The structural identifiability is performed assuming that data for four
% different experimental conditions are available. These conditions are
% defined in ExperimentalConditions_2State.m.
% Copy Transfection_2State.m to this folder
copyfile(fullfile('..','Transfection_2State','Transfection_2State.m'),'Transfection_2State.m');
% Transformation of the model
genssiMultiExperiment('Transfection_2State',...           % Initial model (single
      experiment)
                      'ExperimentalConditions_2State',... % Definition of experimental conditions
                      'Transfection_2State_MultiExp');    % Name of transformed model
% Confirm execution
genssiAskForConfirmation(3);
% Structural identifiability analysis for transformed model
genssiMain('Transfection_2State_MultiExp',3);
```

The result of the conversion is the following (multi-experiment) model:

```
function model = Transfection_2State_MultiExp()
    % Symbolic variables
    syms mRNAExp1 GFPExp1 mRNAExp2 GFPExp2 mRNAExp3 GFPExp3 mRNAExp4 GFPExp4
    syms d b kTL mRNA0
    % Parameters
    model.sym.p = [d;b;kTL;mRNA0];
    % State variables
    model.sym.x = [mRNAExp1;GFPExp1;mRNAExp2;GFPExp2;mRNAExp3;GFPExp3;mRNAExp4;GFPExp4];
    % Control vectors (g)
    model.sym.g = [];
    % Autonomous dynamics (f)
    model.sym.xdot = [-d*mRNAExp1
                      kTL*mRNAExp1 - GFPExp1*b
                      -d*mRNAExp2
                      kTL*mRNAExp2 - GFPExp2*b
                      -d*mRNAExp3
                      (kTL*mRNAExp3)/2 - GFPExp3*b
                      -d*mRNAExp4
                      kTL*mRNAExp4 - (3*GFPExp4*b)/4];
    % Initial conditions
    model.sym.x0 = [mRNA0;0;mRNA0/2;0;mRNA0;0;mRNA0;0];
    % Observables
    model.sym.y = [GFPExp1;GFPExp2;GFPExp3;GFPExp4];
end
```

Based on the original 2 state variables and 4 experiments, we now have 2∗4=8 state variables (model.sym.x). In addition, all parameters now appear directly in the differential equations (model.sym.xdot), and there are no controls.

2.3.4 Transform Model

```
genssiTransformation(modelNameIn,transDef,modelNameOut)
```

genssiTransformation converts a GenSSI model to a new GenSSI model based on a transformation definition.

modelNameIn: the name of the input model (a string).

transDef: the name of a transformation definition file (string).

modelNameOut: the name of the output model (a string).

When we analyze equations for the purpose of determining identifiability, it is sometimes useful to make two changes. The first change is removing redundant equations, which can reduce the number of state variables and the number of parameters. The second change is rescaling the variables, which can reduce the number of parameters. Both of these changes are supported by genssiTransformation.

We begin with a model for mRNA, including translation and degradation. In contrast with the simpler model used for the multi-experiment conversion (above), this model involves mRNA degradation via the action of an enzyme. The model definition is:

```
function model = Transfection_4State()
    % Transfection_2State provides the GenSSI implementation of the 4-state
    % model for mRNA transfection introduced by
    %
    %    Lechtenberg, L. (2015). Model selection in deterministic models of
    %    mRNA transfection. Master Thesis, Ludwig-Maximilians-Universitaet,
    %    Munich, Germany.
    %
    % In contrast to the 2-state transfection model, the 4-state transfection
    % model accounts for an enzymatic degration of the mRNA.
    %
    % The ODE is given by
    %
    %    d[mRNA|/dt      = -d1*[mRNA]-d2*[mRNA]*[enz],      [mRNA](0)      = mRNA0
    %    d[GFP]/dt       = +kTL*[mRNA]-b*[GFP],             [GFP](0)       = 0
    %    d[enz|/dt       = +d3*[mRNA:enz]-d2*[mRNA]*[enz],  [enz](0)       = enz0
    %    d[mRNA:enz]/dt  = -d3*[mRNA:enz]+d2*[mRNA]*[enz],  [mRNA:enz](0)  = 0
    %
    % in which [mRNA], [GFP], [enz] and [mRNA:enz] denote the
    % concentrations of GFP-mRNA, GFP-protein, mRNA degrading enzyme and
    % mRNA-enzyme-complex, respectively.
    % Symbolic variables
    syms mRNA GFP enz mRNAenz
    syms d1 d2 d3 b kTL mRNA0 enz0
    % Parameters
    model.sym.p = [d1;d2;d3;b;kTL;mRNA0;enz0];
    % State variables
    model.sym.x = [mRNA;GFP;enz;mRNAenz];
    % Control vectors (g)
    model.sym.g = [];
    % Autonomous dynamics (f)
    model.sym.xdot = [-d1*mRNA-d2*mRNA*enz
                      +kTL*mRNA-b*GFP
                      +d3*mRNAenz-d2*mRNA*enz
                      -d3*mRNAenz+d2*mRNA*enz];
    % Initial conditions
    model.sym.x0 = [mRNA0;0;enz0;0];
    % Observables
    model.sym.y = [GFP];
end
```

The state variables in this model (model.sym.x) are mRNA, GFP, enzyme (enz), and the mRNA-enzyme complex (mRNAenz). The differential equations show mRNA decreasing due to degradation (-d1∗mRNA) and decreasing due to complexation (-d2∗mRNA∗enz). GFP increases due to translation (kTL∗mRNA) and decreases due to complexation (-d2∗mRNA∗enz). The enzyme (enz) decreases due to complexation (-d2∗mRNA∗enz) and increases due to decomplexation (d3∗mRNAenz). The change in the complex (mRNAenz) is exactly the opposite of the change in the enzyme. As a result of this, we know that enz-enz(0)=-(mRNAenz-mRNAenz(0)) or, since mRNAenz(0)=0, enz-enz0+mRNAenz=0. In addition, we know that the concentration of GFP always depends on the product of mRNA(0)∗kTL, so we will be able to reduce the number of parameters by rescaling (dividing by mRNA0).

With these observations, we can create our transformation definition:

```
function transDef = TransformationRules_4State()
    % TransformationRules_4State defines a state transformation and a
    % parameter substitution for the 4-state model for mRNA transfection
    % introduced by
    %
    %    Lechtenberg, L. (2015). Model selection in deterministic models of
    %    mRNA transfection. Master Thesis, Ludwig-Maximilians-Universitaet,
    %    Munich, Germany.
    %
    % The state transformation reduces the number of state variable by
    % exploiting the conservation relations. The parameter substitution
    % reduced the number of parameters by on, improving the
    % identifiability.
    % Symbolic variables in model
    syms mRNA GFP enz mRNAenz
    syms d1 d2 d3 b kTL mRNA0 enz0
    % Definition of conservation relations
    % (Note: These constraints are set to zeros and used to reduce the
    % dimension of the state space.)
    transDef.sym.constraint = [enz-enz0+mRNAenz];
    % Definition of new state variables
    transDef.sym.state.formula = [mRNA/mRNA0 GFP enz/mRNA0];
    % Definition of new state variables
    transDef.sym.parameter.formula = [d1 d2*mRNA0 d3 b kTL*mRNA0 enz0/mRNA0];
end
```

The transformation is defined by two variables. The first, transDef.sym.Transformation, defines the rescaling. It contains one entry for each element of the final state vector. Since we are converting a model with 4 state variables to 3, this contains 3 elements. The second part of the definition is the constraint, transDef.sym.Constraint. This will be equated to zero during the transformation, so it is enz-enz0+mRNAenz=0, or, equivalently, enz-enz(0)=-(mRNAenz-mRNAenz(0)). The variable transDef.sym.Constraint can contain multiple constraints, separated by a semicolon. Finally, there are two optional definitions of substitutions. They contain a variable number of rows, each of which is a substitution, or change of names. During the transformation process, new variables are created for the state vector and the parameters, and the names are changed via the rules "*"->"_times_" (for "times") and "/"->"_div_" (for "divided by"). These names can be considered as suggestions for the new names, and the user can override them with the stateSubs and parSubs definitions. For example, mRNA_div_mRNA0 is replaced by mnew in the state vector, and d2*mRNA0 is replaced by d2_times_mRNA0 in the parameters. It is considered good practice to leave these 2 definitions out the first time the transformation is run, and then add them in later in order to gain more control over the naming.

This transformation is started by running the following line of code:

```
runTransfection_4State_Transformation

genssiTransformation('Transfection_4State','TransformationRules_4State','
      Transfection_4State_Transformation');

% runTransfection_4State_Transformation performs the structural
% identifiability analysis for the 4-state model for mRNA transfection
% introduced by
%
%    Lechtenberg, L. (2015). Model selection in deterministic models of
%    mRNA transfection. Master Thesis, Ludwig-Maximilians-Universitaet,
%    Munich, Germany.
%
% In contrast to the 2-state transfection model, the 4-state transfection
% model accounts for an enzymatic degration of the mRNA.
%
% Here the model is transformed using informatin provided in
% TransformationRules_4State.m. The state transformation reduces the number
% of state variable by exploiting the conservation relations. The parameter
% substitution reduced the number of parameters by on, improving the
% identifiability.
% Copy Transfection_4State.m to this folder
copyfile(fullfile('..','Transfection_4State','Transfection_4State.m'),'Transfection_4State.m');
% Transformation of the model
genssiTransformation('Transfection_4State',...          % Initial model
                     'TransformationRules_4State',...    % Definition of transformation
                     'Transfection_4State_Transformation'); % Name of transformed model
% Confirm execution
genssiAskForConfirmation(3);
% Structural identifiability analysis for transformed model
genssiMain('Transfection_4State_Transformation',7);
```

The result of the transformation is the following new model:

```
function model = Transfection_4State_Transformation()
    % Symbolic variables
    syms mRNA_div_mRNA0 GFP enz_div_mRNA0
    syms d1 d2_times_mRNA0 d3 b kTL_times_mRNA0 enz0_div_mRNA0
    % Parameters
    model.sym.p = [d1;d2_times_mRNA0;d3;b;kTL_times_mRNA0;enz0_div_mRNA0];
    % State variables
    model.sym.x = [mRNA_div_mRNA0;GFP;enz_div_mRNA0];
    % Control vectors (g)
    model.sym.g = [];
    % Autonomous dynamics (f)
    model.sym.xdot = [-mRNA_div_mRNA0*(d1 + d2_times_mRNA0*enz_div_mRNA0)
                      kTL_times_mRNA0*mRNA_div_mRNA0 - GFP*b
                      d3*enz0_div_mRNA0 - d3*enz_div_mRNA0 - d2_times_mRNA0*enz_div_mRNA0*mRNA_div_mRNA0];
    % Initial conditions
    model.sym.x0 = [1;0;enz0_div_mRNA0];
    % Observables
    model.sym.y = [GFP];
end
```

In this transformed model, the new state variables are mnew, Gnew, and E1new, based on the definition transDef.stateSubs. We could just as well have left the names of mRNA_div_mRNA0, GFP, and enz_div_mRNA0, or used the simpler names of mRNA, GFP, and enz for the new state vector. A similar remark is valid for the parameter names. The resulting differential equations (model.sym.xdot) are less readable than in the original model, but we have eliminated one state variable and one parameter.

3 Code Organization

In the following we will briefly outline how the GenSSI code is organized. For a more detailed description we refer the reader to the documentation of the individual functions.

3.1 Directory Structure

The main, or root, directory, which we refer to as GenSSI, contains the GenSSI functions which the user is likely to call directly. In addition, the following subdirectories are used:

- GenSSI/Auxiliary contains the main logic and some auxiliary functions, such as genssiRemoveZeroRows.
- GenSSI/Examples contains GenSSI model definitions and the results of analysis.
- GenSSI/Docu contains tools for creating the GenSSI documentation, as well as input and output of that process.
- GenSSI/Docu/config contains configuration files for the documentation tools.
- GenSSI/Docu/input contains input for document creation, including .dox files.
- GenSSI/Docu/output contains the output of document creation.

3.2 Document Creation

New versions of the documentation are created with the help of:

- MatlabDocMaker.m (in GenSSI/Docu)
- mtoc++ (needs to be installed and available via the path variable)
- Doxygen (needs to be installed and available via the path variable)
- LaTex (needs to be installed and available via the path variable)
- Graphviz (needs to be installed and available via the path variable)
- Ghostscript (needs to be installed and available via the path variable)

The documentation configuration is changed by editing the files in the GenSSI/Docu/config directory and by running

```
MatlabDocMaker.setup
```

A new version of the documentation is created by calling

```
MatlabDocMaker.create('latex',true)
```

This results in an html version of the guide (index.html and many other files in GenSSI/Docu/output), and a pdf version (refman.pdf in GenSSI/Docu/output/latex).

4 Hierarchical Index

4.1 Class Hierarchy

This inheritance list is sorted roughly, but not completely, alphabetically:

handle

SBMLode 14

5 Class Index

5.1 Class List

Here are the classes, structs, unions and interfaces with brief descriptions:

SBMLode
SBMLMODEL provides an intermediate container between the SBML definition and an amimodel object 14

6 Class Documentation

6.1 SBMLode Class Reference

SBMLMODEL provides an intermediate container between the SBML definition and an amimodel object.

Inheritance diagram for SBMLode:

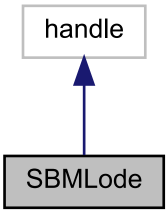

Collaboration diagram for SBMLode:

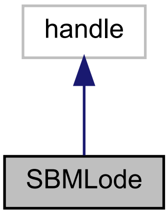

Public Member Functions

- SBMLode (matlabtypesubstitute filename)

  SBMLode extracts information from an SBML definition and stores it in a symbolic format.
- noret::substitute importSBML (matlabtypesubstitute filename)

  importSBML parses information from the SBML definition and populates the SBMLode object from this information.
- noret::substitute checkODE ()

  checkODE checks whether the length of various variable names exceeds namelengthmax (would cause troube with symbolic processing later on).
- noret::substitute writeAMICI (matlabtypesubstitute modelname)

  writeAMICI writes the symbolic information from an SBMLode object into an AMICI model definition file

Public Attributes

- matlabtypesubstitute state = sym.empty("")

  states
- matlabtypesubstitute observable = sym.empty("")

  observables
- matlabtypesubstitute observable_name = sym.empty("")

  names of observables
- matlabtypesubstitute param = sym.empty("")

  parameter names
- matlabtypesubstitute parameter = sym.empty("")

  parameter expressions
- matlabtypesubstitute constant = sym.empty("")

  constants
- matlabtypesubstitute reaction = sym.empty("")

  reactions
- matlabtypesubstitute compartment = sym.empty("")

  compartments
- matlabtypesubstitute volume = sym.empty("")

  compartment volumes
- matlabtypesubstitute kvolume = sym.empty("")

  condition volumes
- matlabtypesubstitute initState = sym.empty("")

initial condition of states
- matlabtypesubstitute condition = sym.empty("")

  condition
- matlabtypesubstitute flux = sym.empty("")

  reaction fluxes
- matlabtypesubstitute stochiometry = sym.empty("")

  reaction stochiometry
- matlabtypesubstitute xdot = sym.empty("")

  right hand side of reconstructed differential equation
- matlabtypesubstitute trigger = sym.empty("")

  event triggers
- matlabtypesubstitute bolus = sym.empty("")

  event boli
- matlabtypesubstitute funmath = cell.empty("")

  mathematical experessions for function
- matlabtypesubstitute funarg = cell.empty("")

  string function signature
- matlabtypesubstitute time_symbol = char.empty("")

  symbol of time
- matlabtypesubstitute pnom = double.empty("")

  nominal parameters
- matlabtypesubstitute knom = double.empty("")

  nominal conditions

6.1.1 Detailed Description

Definition at line 17 of file SBMLode.m.

6.1.2 Constructor & Destructor Documentation

6.1.2.1 SBMLode()

```
SBMLode (
            matlabtypesubstitute filename )
```

Parameters

filename	target name of the model (excluding the suffix .xml/.sbml)

Definition at line 207 of file SBMLode.m.

Here is the call graph for this function:

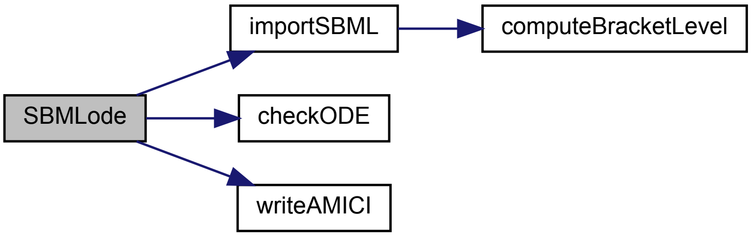

6.1.3 Member Function Documentation

6.1.3.1 importSBML()

```
noret::substitute importSBML (
            matlabtypesubstitute filename )
```

Parameters

filename	target name of the model

Return values

filename	void

Definition at line 18 of file importSBML.m.

Here is the call graph for this function:

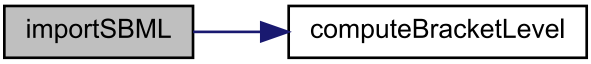

Here is the caller graph for this function:

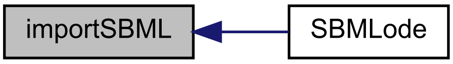

6.1.3.2 checkODE()

```
noret::substitute checkODE ( )
```

Return values

void	

Definition at line 18 of file checkODE.m.

Here is the caller graph for this function:

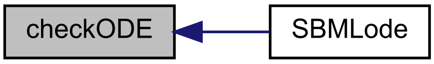

6.1.3.3 writeAMICI()

```
noret::substitute writeAMICI (
            matlabtypesubstitute modelname )
```

Parameters

modelname	target name of the model (_syms.m will be appended to the name)

Return values

modelname	void

Definition at line 18 of file writeAMICI.m.

Here is the caller graph for this function:

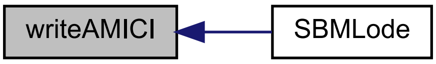

6.1.4 Member Data Documentation

6.1.4.1 state

```
state = sym.empty("")
```

Default: sym.empty("")

Definition at line 28 of file SBMLode.m.

6.1.4.2 observable

```
observable = sym.empty("")
```

Default: sym.empty("")

Definition at line 36 of file SBMLode.m.

6.1.4.3 observable_name

```
observable_name = sym.empty("")
```

Default: sym.empty("")

Definition at line 44 of file SBMLode.m.

6.1.4.4 param

```
param = sym.empty("")
```

Default: sym.empty("")

Definition at line 52 of file SBMLode.m.

6.1.4.5 parameter

```
parameter = sym.empty("")
```

Default: sym.empty("")

Definition at line 60 of file SBMLode.m.

6.1.4.6 constant

```
constant = sym.empty("")
```

Default: sym.empty("")

Definition at line 68 of file SBMLode.m.

6.1.4.7 reaction

```
reaction = sym.empty("")
```

Default: sym.empty("")

Definition at line 76 of file SBMLode.m.

6.1.4.8 compartment

```
compartment = sym.empty("")
```

Default: sym.empty("")

Definition at line 84 of file SBMLode.m.

6.1.4.9 volume

```
volume = sym.empty("")
```

Default: sym.empty("")

Definition at line 92 of file SBMLode.m.

6.1.4.10 kvolume

```
kvolume = sym.empty("")
```

Default: sym.empty("")

Definition at line 100 of file SBMLode.m.

6.1.4.11 initState

```
initState = sym.empty("")
```

Default: sym.empty("")

Definition at line 108 of file SBMLode.m.

6.1.4.12 condition

```
condition = sym.empty("")
```

Default: sym.empty("")

Definition at line 116 of file SBMLode.m.

6.1.4.13 flux

```
flux = sym.empty("")
```

Default: sym.empty("")

Definition at line 124 of file SBMLode.m.

6.1.4.14 stochiometry

```
stochiometry = sym.empty("")
```

Default: sym.empty("")

Definition at line 132 of file SBMLode.m.

6.1.4.15 xdot

```
xdot = sym.empty("")
```

Default: sym.empty("")

Definition at line 140 of file SBMLode.m.

6.1.4.16 trigger

```
trigger = sym.empty("")
```

Default: sym.empty("")

Definition at line 148 of file SBMLode.m.

6.1.4.17 bolus

```
bolus = sym.empty("")
```

Default: sym.empty("")

Definition at line 156 of file SBMLode.m.

6.1.4.18 funmath

```
funmath = cell.empty("")
```

Default: cell.empty("")

Definition at line 164 of file SBMLode.m.

6.1.4.19 funarg

```
funarg = cell.empty("")
```

Default: cell.empty("")

Definition at line 172 of file SBMLode.m.

6.1.4.20 time_symbol

```
time_symbol = char.empty("")
```

Default: char.empty("")

Definition at line 180 of file SBMLode.m.

6.1.4.21 pnom

```
pnom = double.empty("")
```

Default: double.empty("")

Definition at line 188 of file SBMLode.m.

6.1.4.22 knom

```
knom = double.empty("")
```

Default: double.empty("")

Definition at line 196 of file SBMLode.m.

7 File Documentation

7.1 Auxiliary/amiciStructToSource.m File Reference

amiciStructToSource converts a model definition (struct) to a source format (MATLAB function file) and saves the results in the examples directory.

Functions

- noret::substitute amiciStructToSource (matlabtypesubstitute model)

  amiciStructToSource converts a model definition (struct) to a source format (MATLAB function file) and saves the results in the examples directory.

7.1.1 Function Documentation

7.1.1.1 amiciStructToSource()

```
noret::substitute amiciStructToSource (
            matlabtypesubstitute model )
```

Parameters

model	model definition (struct)

Return values

model	void

Definition at line 17 of file amiciStructToSource.m.

7.2 Auxiliary/genssiAskForConfirmation.m File Reference

genssiAskForConfirmation informs the user about the expected runtime for an example and asks the user to confirm that the example is executed.

Functions

- noret::substitute genssiAskForConfirmation (matlabtypesubstitute expectedRuntime)
  genssiAskForConfirmation informs the user about the expected runtime for an example and asks the user to confirm that the example is executed.

7.2.1 Function Documentation

7.2.1.1 genssiAskForConfirmation()

```
noret::substitute genssiAskForConfirmation (
            matlabtypesubstitute expectedRuntime )
```

Parameters

expectedRuntime	expected runtime in seconds

Return values

expectedRuntime	void

Definition at line 17 of file genssiAskForConfirmation.m.

Here is the call graph for this function:

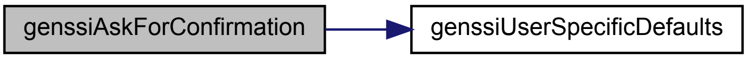

7.3 Auxiliary/genssiCheckExpCondition.m File Reference

genssiCheckExpCondition checks the consistency of a GenSSI experiment condition for a given model.

Functions

- mlhsInnerSubst< matlabtypesubstitute > genssiCheckExpCondition (matlabtypesubstitute model, matlabtypesubstitute expCond)

  genssiCheckExpCondition checks the consistency of a GenSSI experiment condition for a given model.

7.3.1 Function Documentation

7.3.1.1 genssiCheckExpCondition()

```
mlhsInnerSubst< matlabtypesubstitute > genssiCheckExpCondition (
            matlabtypesubstitute model,
            matlabtypesubstitute expCond )
```

Parameters

model	GenSSI model
expCond	GenSSI experiment condition

Return values

expCond	GenSSI experiment condition

Definition at line 17 of file genssiCheckExpCondition.m.

Here is the call graph for this function:

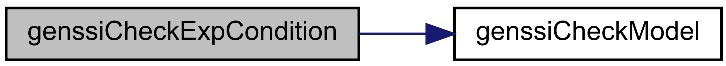

Here is the caller graph for this function:

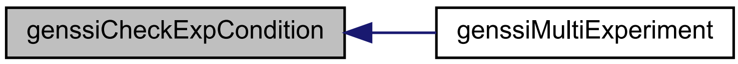

7.4 Auxiliary/genssiCheckModel.m File Reference

genssiCheckModel checks the consistency of a GenSSI model. It compares the dimensions of different entries and transforms them if possible.

Functions

- mlhsInnerSubst< matlabtypesubstitute > genssiCheckModel (matlabtypesubstitute model)

  genssiCheckModel checks the consistency of a GenSSI model. It compares the dimensions of different entries and transforms them if possible.

7.4.1 Function Documentation

7.4.1.1 genssiCheckModel()

```
mlhsInnerSubst< matlabtypesubstitute > genssiCheckModel (
            matlabtypesubstitute model )
```

Parameters

model	GenSSI model

Return values

model	GenSSI model

Definition at line 17 of file genssiCheckModel.m.

Here is the caller graph for this function:

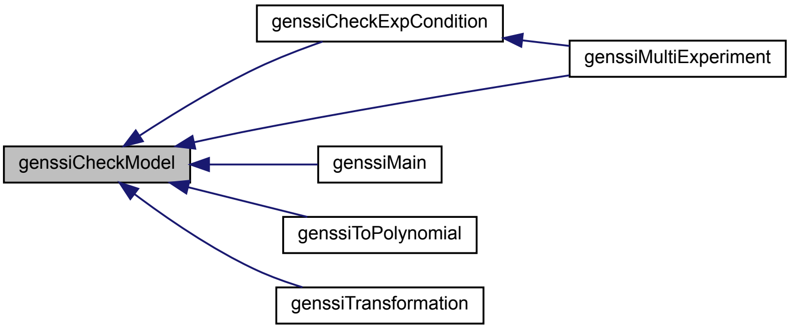

7.5 Auxiliary/genssiComputeLieDerivatives.m File Reference

genssiComputeLieDerivatives computes Lie derivatives of the output functions (transpose(model.sym.y)), the state vectors (model.sym.x), and the initial conditions (transpose(model.sym.x0)) with respect to the equations (transpose(model.sym.xdot)) and controls (transpose(model.sym.g))

Functions

- mlhsSubst< mlhsInnerSubst< matlabtypesubstitute >,mlhsInnerSubst< matlabtypesubstitute > > genssiComputeLieDerivatives (matlabtypesubstitute model, matlabtypesubstitute options)

  genssiComputeLieDerivatives computes Lie derivatives of the output functions (transpose(model.sym.y)), the state vectors (model.sym.x), and the initial conditions (transpose(model.sym.x0)) with respect to the equations (transpose(model.sym.xdot)) and controls (transpose(model.sym.g))
- mlhsSubst< mlhsInnerSubst< matlabtypesubstitute >,mlhsInnerSubst< matlabtypesubstitute > > mtoc_subst_genssiComputeLieDerivatives_m_tsbus_cotm_jacRank (matlabtypesubstitute LDer, matlabtypesubstitute rankVector, matlabtypesubstitute order, matlabtypesubstitute model, matlabtypesubstitute options)

  jacRank computes jacobian and rank, producing output text

7.5.1 Function Documentation

7.5.1.1 genssiComputeLieDerivatives()

```
mlhsSubst< mlhsInnerSubst< matlabtypesubstitute >,mlhsInnerSubst< matlabtypesubstitute > >
genssiComputeLieDerivatives (
            matlabtypesubstitute model,
            matlabtypesubstitute options )
```

Parameters

model	model definition (struct)
options	processing options (struct)

Return values

options	processing options (struct)
VectorLieDerivatives	a vector of all Lie derivatives

Definition at line 17 of file genssiComputeLieDerivatives.m.

Here is the call graph for this function:

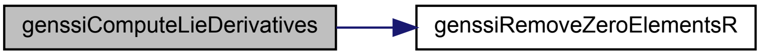

Here is the caller graph for this function:

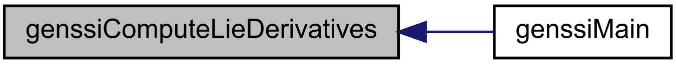

7.5.1.2 mtoc_subst_genssiComputeLieDerivatives_m_tsbus_cotm_jacRank()

```
mlhsSubst< mlhsInnerSubst< matlabtypesubstitute >,mlhsInnerSubst< matlabtypesubstitute > >
mtoc_subst_genssiComputeLieDerivatives_m_tsbus_cotm_jacRank (
            matlabtypesubstitute LDer,
            matlabtypesubstitute rankVector,
            matlabtypesubstitute order,
            matlabtypesubstitute model,
            matlabtypesubstitute options )
```

Parameters

LDer	model definition (struct)
rankVector	vector of ranks (for results)
order	for output text, computing derivatives of order
model	model definition
options	processing options (struct)

Return values

rankFull	boolean, true if rank is full
rankVector	vector of ranks (for results)

Definition at line 116 of file genssiComputeLieDerivatives.m.

7.6 Auxiliary/genssiComputeReducedTableau.m File Reference

genssiComputeReducedTableau computes reduced tableaus of the jacobian by eliminating rows and columns where solutions to relationships can found or excluded.

Functions

- mlhsSubst< mlhsInnerSubst< matlabtypesubstitute >,mlhsInnerSubst< matlabtypesubstitute >,mlhsInnerSubst< matlabtypesubstitute >,mlhsInnerSubst< matlabtypesubstitute >,mlhsInnerSubst< matlabtypesubstitute > > genssiComputeReducedTableau (matlabtypesubstitute model, matlabtypesubstitute results, matlabtypesubstitute VectorLieDerivatives, matlabtypesubstitute JacParam, matlabtypesubstitute options)

  genssiComputeReducedTableau computes reduced tableaus of the jacobian by eliminating rows and columns where solutions to relationships can found or excluded.

7.6.1 Function Documentation

7.6.1.1 genssiComputeReducedTableau()

```
mlhsSubst< mlhsInnerSubst< matlabtypesubstitute >,mlhsInnerSubst< matlabtypesubstitute >,mlhsInnerSubst<
matlabtypesubstitute >,mlhsInnerSubst< matlabtypesubstitute >,mlhsInnerSubst< matlabtypesubstitute
> > genssiComputeReducedTableau (
            matlabtypesubstitute model,
            matlabtypesubstitute results,
            matlabtypesubstitute VectorLieDerivatives,
            matlabtypesubstitute JacParam,
            matlabtypesubstitute options )
```

Parameters

model	model definition (struct)
results	results of compute tableau (symbolic matrix)
VectorLieDerivatives	vector of Lie derivatives (symbolic array)
JacParam	jacobian with respect to parameters (symbolic matrix)
options	options (struct)

Return values

options	options (struct)
results	results of compute tableau (symbolic matrix)

Return values

RJacParam01	reduced tableau (binary matrix)
ECC	equations (symbolic matrix)
rParam	reduced list of parameters (symbolic array)

Definition at line 17 of file genssiComputeReducedTableau.m.

Here is the call graph for this function:

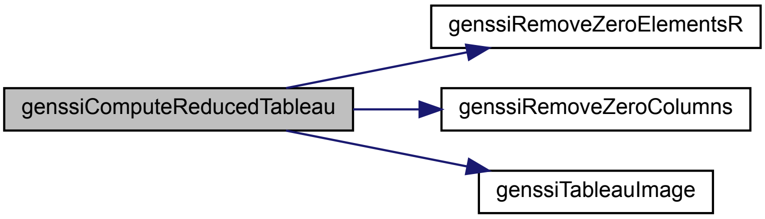

Here is the caller graph for this function:

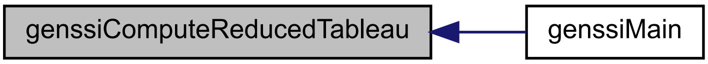

7.7 Auxiliary/genssiComputeTableau.m File Reference

genssiComputeTableau computes the tableau based on the jacobian of the Lie derivatives.

Functions

- mlhsSubst< mlhsInnerSubst< matlabtypesubstitute >,mlhsInnerSubst< matlabtypesubstitute >,mlhsInnerSubst< matlabtypesubstitute > > genssiComputeTableau (matlabtypesubstitute model, matlabtypesubstitute VectorLieDerivatives, matlabtypesubstitute options)

    genssiComputeTableau computes the tableau based on the jacobian of the Lie derivatives.

7.7.1 Function Documentation

7.7.1.1 genssiComputeTableau()

```
mlhsSubst< mlhsInnerSubst< matlabtypesubstitute >,mlhsInnerSubst< matlabtypesubstitute >,mlhsInnerSubst<
matlabtypesubstitute > > genssiComputeTableau (
            matlabtypesubstitute model,
            matlabtypesubstitute VectorLieDerivatives,
            matlabtypesubstitute options )
```

Parameters

model	model definition (struct)
VectorLieDerivatives	vector of Lie derivatives (symbolic array)
options	options (struct)

Return values

options	options (struct)
results	results of calculations (struct)
JacParam	jacobian of the Lie derivatives with respect to the parameters (symbolic matrix)

Definition at line 17 of file genssiComputeTableau.m.

Here is the call graph for this function:

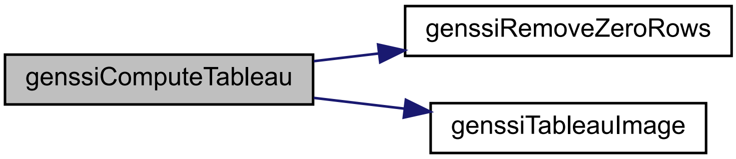

Here is the caller graph for this function:

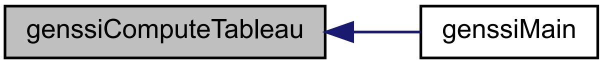

7.8 Auxiliary/genssiGetCandForTransformation.m File Reference

genssiGetCandForTransformation provides candidates for a reduced parameterisation of a model

Functions

- mlhsInnerSubst< matlabtypesubstitute > genssiGetCandForTransformation (matlabtypesubstitute model)

  genssiGetCandForTransformation provides candidates for a reduced parameterisation of a model

7.8.1 Function Documentation

7.8.1.1 genssiGetCandForTransformation()

```
mlhsInnerSubst< matlabtypesubstitute > genssiGetCandForTransformation (
            matlabtypesubstitute model )
```

Parameters

model	GenSSI model

Return values

cand	vector of candidates

Definition at line 17 of file genssiGetCandForTransformation.m.

Here is the caller graph for this function:

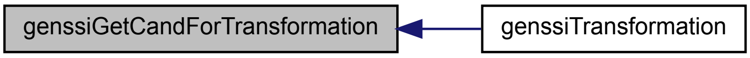

7.9 Auxiliary/genssiGetSymChar.m File Reference

genssiGetSymChar converts a char into an other char which can be used as name of a symbolic variable. Therefore, matheamtical operatores ($+$, $-$, $*$, $/$ and $^\wedge$) are replaced by strings.

Functions

- mlhsInnerSubst< matlabtypesubstitute > genssiGetSymChar (matlabtypesubstitute str)

  genssiGetSymChar converts a char into an other char which can be used as name of a symbolic variable. Therefore, matheamtical operatores ($+$, $-$, $*$, $/$ and $^\wedge$) are replaced by strings.

7.9.1 Function Documentation

7.9.1.1 genssiGetSymChar()

```
mlhsInnerSubst< matlabtypesubstitute > genssiGetSymChar (
            matlabtypesubstitute str )
```

Parameters

str	a char

Return values

str	a char

Definition at line 17 of file genssiGetSymChar.m.

Here is the caller graph for this function:

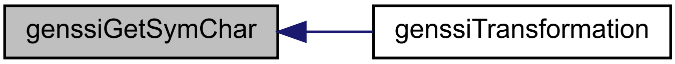

7.10 Auxiliary/genssiOrderTableau.m File Reference

genssiOrderTableau orders tableaus, searches for new opportunities to eliminate rows or columns be solving equations, and creates new (reduced) tableaus.

Functions

- mlhsSubst< mlhsInnerSubst< matlabtypesubstitute >,mlhsInnerSubst< matlabtypesubstitute > > genssiOrderTableau (matlabtypesubstitute model, matlabtypesubstitute results, matlabtypesubstitute RJacParam01, matlabtypesubstitute ECC, matlabtypesubstitute rParam, matlabtypesubstitute options)

  genssiOrderTableau orders tableaus, searches for new opportunities to eliminate rows or columns be solving equations, and creates new (reduced) tableaus.
- mlhsSubst< mlhsInnerSubst< matlabtypesubstitute >,mlhsInnerSubst< matlabtypesubstitute >,mlhsInnerSubst< matlabtypesubstitute >,mlhsInnerSubst< matlabtypesubstitute >,mlhsInnerSubst< matlabtypesubstitute >,mlhsInnerSubst< matlabtypesubstitute >,mlhsInnerSubst< matlabtypesubstitute > > mtoc_subst_genssiOrderTableau_m_ (matlabtypesubstitute Param, matlabtypesubstitute Param_local, matlabtypesubstitute global_ident_par, matlabtypesubstitute Mat_index, matlabtypesubstitute RJacparam_new, matlabtypesubstitute RJacParam01_nonzero_rows, matlabtypesubstitute sum_RJacParam01_nonzero_rows_t, matlabtypesubstitute ECC, matlabtypesubstitute ECC_new, matlabtypesubstitute options)

  displayRelevantParameters displays relevant parameters
- mlhsSubst< mlhsInnerSubst< matlabtypesubstitute >,mlhsInnerSubst< matlabtypesubstitute >,mlhsInnerSubst< matlabtypesubstitute >,mlhsInnerSubst< matlabtypesubstitute >,mlhsInnerSubst< matlabtypesubstitute >,mlhsInnerSubst< matlabtypesubstitute > > mtoc_subst_genssiOrderTableau_m_tsbus_cotm_displayReducedTableau (matlabtypesubstitute ECC_remaining, matlabtypesubstitute Param_local, matlabtypesubstitute Param_display, matlabtypesubstitute global_ident_par, matlabtypesubstitute display_tableau_RJacparam_new, matlabtypesubstitute number_fig, matlabtypesubstitute options)

displayReducedTableau displays reduced tableaus

- mlhsSubst< mlhsInnerSubst< matlabtypesubstitute >,mlhsInnerSubst< matlabtypesubstitute > > mtoc_subst_genssiOrderTableau_m_tsbus_cotm_displayRemainingParameters (matlabtypesubstitute ECC_remaining, matlabtypesubstitute Param_local, matlabtypesubstitute Param_remaining, matlabtypesubstitute global_ident_par, matlabtypesubstitute display_tableau_RJacparam_new, matlabtypesubstitute row_index_1, matlabtypesubstitute tableau_for_second_reduced_tableau, matlabtypesubstitute parameters_for_second_reduced_tableau, matlabtypesubstitute number_fig, matlabtypesubstitute options)

  displayReducedTableau displays the remaining parameters
- mlhsSubst< mlhsInnerSubst< matlabtypesubstitute >,mlhsInnerSubst< matlabtypesubstitute > > mtoc_subst_genssiOrderTableau_m_tsbus_cotm_solveRemPar (matlabtypesubstitute ECC, matlabtypesubstitute Param, matlabtypesubstitute Param_local, matlabtypesubstitute global_ident_par)

  solveRemPar solves the remaining parameters
- mlhsSubst< mlhsInnerSubst< matlabtypesubstitute >,mlhsInnerSubst< matlabtypesubstitute > > mtoc_subst_genssiOrderTableau_m_tsbus_cotm_getIndexOfDuplicateParams (matlabtypesubstitute ECC, matlabtypesubstitute RJacParam01_nonzero_rows)

  getIndexOfDuplicateParams gets index of duplicate parameters

7.10.1 Function Documentation

7.10.1.1 genssiOrderTableau()

```
mlhsSubst< mlhsInnerSubst< matlabtypesubstitute >,mlhsInnerSubst< matlabtypesubstitute > >
genssiOrderTableau (
            matlabtypesubstitute model,
            matlabtypesubstitute results,
            matlabtypesubstitute RJacParam01,
            matlabtypesubstitute ECC,
            matlabtypesubstitute rParam,
            matlabtypesubstitute options )
```

Parameters

model	model definition (struct)
results	results of previous steps (struct)
RJacParam01	reduced tableau, i.e. binary form of jacobian of the Lie derivatives with respect to the parameters (binary matrix)
ECC	equations (symbolic array)
rParam	reduced list of parameters (symbolic array)
options	options (struct)

Return values

options	options (struct)
results	results of previous steps (struct)

Definition at line 17 of file genssiOrderTableau.m.

Here is the call graph for this function:

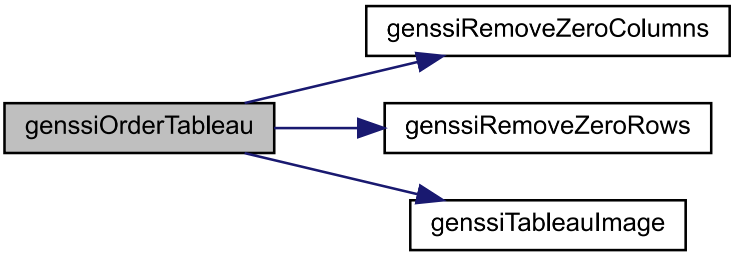

Here is the caller graph for this function:

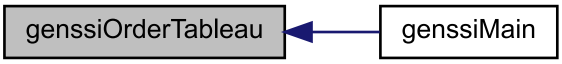

7.11 Auxiliary/genssiPolySys.m File Reference

genssiPolySys converts a model to polynomial form. [z,fz,gz,z0,yz,xi,inv_xi] = genssiPolySys(x,f,g,x0,y)

Functions

- mlhsSubst< mlhsInnerSubst< matlabtypesubstitute >,mlhsInnerSubst< matlabtypesubstitute >,mlhsInnerSubst< matlabtypesubstitute >,mlhsInnerSubst< matlabtypesubstitute >,mlhsInnerSubst< matlabtypesubstitute >,mlhsInnerSubst< matlabtypesubstitute >,mlhsInnerSubst< matlabtypesubstitute > > genssiPolySys (matlabtypesubstitute x, matlabtypesubstitute f, matlabtypesubstitute g, matlabtypesubstitute x0, matlabtypesubstitute y)

  genssiPolySys converts a model to polynomial form. [z,fz,gz,z0,yz,xi,inv_xi] = genssiPolySys(x,f,g,x0,y)

7.11.1 Function Documentation

7.11.1.1 genssiPolySys()

```
mlhsSubst< mlhsInnerSubst< matlabtypesubstitute >,mlhsInnerSubst< matlabtypesubstitute >,mlhsInnerSubst<
matlabtypesubstitute >,mlhsInnerSubst< matlabtypesubstitute >,mlhsInnerSubst< matlabtypesubstitute
>,mlhsInnerSubst< matlabtypesubstitute >,mlhsInnerSubst< matlabtypesubstitute > > genssiPolySys
(
            matlabtypesubstitute x,
            matlabtypesubstitute f,
            matlabtypesubstitute g,
            matlabtypesubstitute x0,
            matlabtypesubstitute y )
```

Parameters

x	the state variables of the input model (a vector, nx x 1)
f	the vector field of the autonomous system of the input model (a vector, nx x 1)
g	the control matrix of the input model (a matrix, nx x nu)
x0	the initial conditions of the input model (a vector, nx x 1)
y	the output variables of the input model (a vector, ny x 1)

Return values

z	the state variables of the transformed model (a vector, nz x 1)
fz	the vector field of the autonomous system of the transformed model (a vector, nz x 1)
gz	the control matrix of the transformed model (a matrix, nz x nu)
z0	the initial conditions of the transformed model (a vector, nz x 1)
yz	the output variables of the transformed model (a vector, ny x 1)
xi	the set of denominators
inv_xi	the set of unique denominators

Definition at line 17 of file genssiPolySys.m.

Here is the caller graph for this function:

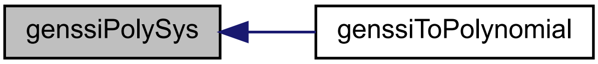

7.12 Auxiliary/genssiRemoveZeroColumns.m File Reference

genssiRemoveZeroColumns removes zero columns from a matrix

Functions

- mlhsSubst< mlhsInnerSubst< matlabtypesubstitute >,mlhsInnerSubst< matlabtypesubstitute >,mlhsInnerSubst< matlabtypesubstitute > > genssiRemoveZeroColumns (matlabtypesubstitute matrixIn)

  genssiRemoveZeroColumns removes zero columns from a matrix

7.12.1 Function Documentation

7.12.1.1 genssiRemoveZeroColumns()

```
mlhsSubst< mlhsInnerSubst< matlabtypesubstitute >,mlhsInnerSubst< matlabtypesubstitute >,mlhsInnerSubst<
matlabtypesubstitute > > genssiRemoveZeroColumns (
            matlabtypesubstitute matrixIn )
```

Parameters

matrixIn	input (matrix)

Return values

matrixOut	output (matrix)
keepBoolean	boolean vector of indices kept (array)
keepIndex	vector of indices keept (array)

Definition at line 17 of file genssiRemoveZeroColumns.m.

Here is the caller graph for this function:

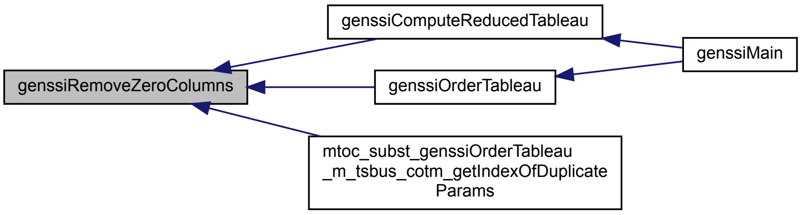

7.13 Auxiliary/genssiRemoveZeroElementsC.m File Reference

genssiRemoveZeroElements removes zero columns from a row vecor

Functions

- mlhsSubst< mlhsInnerSubst< matlabtypesubstitute >,mlhsInnerSubst< matlabtypesubstitute >,mlhsInnerSubst< matlabtypesubstitute > > genssiRemoveZeroElementsC (matlabtypesubstitute vectorIn)

    genssiRemoveZeroElements removes zero columns from a row vecor

7.13.1 Function Documentation

7.13.1.1 genssiRemoveZeroElementsC()

```
mlhsSubst< mlhsInnerSubst< matlabtypesubstitute >,mlhsInnerSubst< matlabtypesubstitute >,mlhsInnerSubst<
matlabtypesubstitute > > genssiRemoveZeroElementsC (
            matlabtypesubstitute vectorIn )
```

Parameters

vectorIn	input (array)

Return values

vectorOut	output (array)
keepBoolean	boolean vector of indices kept (array)
keepIndex	vector of indices kept (array)

Definition at line 17 of file genssiRemoveZeroElementsC.m.

Here is the caller graph for this function:

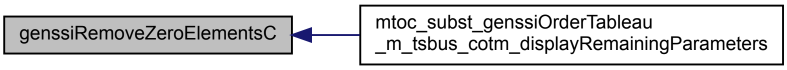

7.14 Auxiliary/genssiRemoveZeroElementsR.m File Reference

genssiRemoveZeroElements removes zero columns from a row vecor

Functions

- mlhsSubst< mlhsInnerSubst< matlabtypesubstitute >,mlhsInnerSubst< matlabtypesubstitute >,mlhsInnerSubst< matlabtypesubstitute > > genssiRemoveZeroElementsR (matlabtypesubstitute vectorIn)

    genssiRemoveZeroElements removes zero columns from a row vecor

7.14.1 Function Documentation

7.14.1.1 genssiRemoveZeroElementsR()

```
mlhsSubst< mlhsInnerSubst< matlabtypesubstitute >,mlhsInnerSubst< matlabtypesubstitute >,mlhsInnerSubst<
matlabtypesubstitute > > genssiRemoveZeroElementsR (
            matlabtypesubstitute vectorIn )
```

Parameters

vectorIn	input (array)

Return values

vectorOut	output (array)
keepBoolean	boolean vector of indices kept (array)
keepIndex	vector of indices kept (array)

Definition at line 17 of file genssiRemoveZeroElementsR.m.

Here is the caller graph for this function:

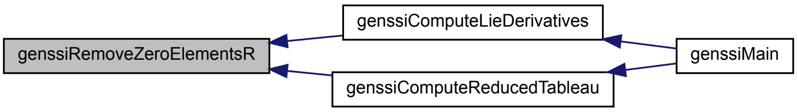

7.15 Auxiliary/genssiRemoveZeroRows.m File Reference

genssiRemoveZeroRows removes zero rows from a matrix

Functions

- mlhsSubst< mlhsInnerSubst< matlabtypesubstitute >,mlhsInnerSubst< matlabtypesubstitute >,mlhsInnerSubst< matlabtypesubstitute > > genssiRemoveZeroRows (matlabtypesubstitute matrixIn)

    genssiRemoveZeroRows removes zero rows from a matrix

7.15.1 Function Documentation

7.15.1.1 genssiRemoveZeroRows()

```
mlhsSubst< mlhsInnerSubst< matlabtypesubstitute >,mlhsInnerSubst< matlabtypesubstitute >,mlhsInnerSubst<
matlabtypesubstitute > > genssiRemoveZeroRows (
            matlabtypesubstitute matrixIn )
```

Parameters

matrixIn	input (matrix)

Return values

matrixOut	output (matrix)
keepBoolean	boolean vector of indices kept (array)
keepIndex	vector of indices keept (array)

Definition at line 17 of file genssiRemoveZeroRows.m.

Here is the caller graph for this function:

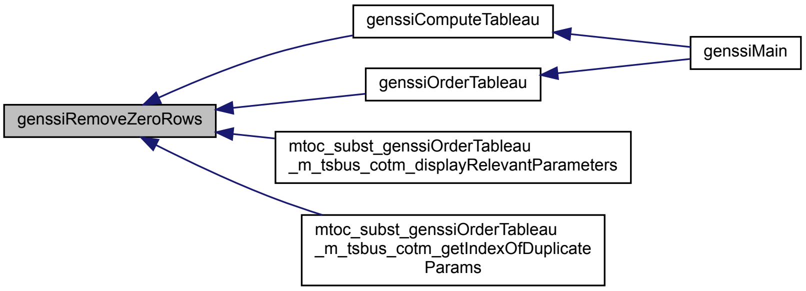

7.16 Auxiliary/genssiReportInputs.m File Reference

genssiReportInputs reports inputs, i.e. model definition.

Functions

- mlhsInnerSubst< matlabtypesubstitute > genssiReportInputs (matlabtypesubstitute model, matlabtypesubstitute options)

  genssiReportInputs reports inputs, i.e. model definition.

7.16.1 Function Documentation

7.16.1.1 genssiReportInputs()

```
mlhsInnerSubst< matlabtypesubstitute > genssiReportInputs (
            matlabtypesubstitute model,
            matlabtypesubstitute options )
```

Parameters

model	model definition (struct)
options	options (struct)

Return values

options	options (struct)

Definition at line 17 of file genssiReportInputs.m.

Here is the caller graph for this function:

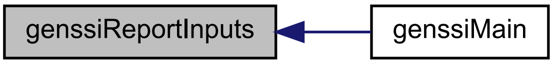

7.17 Auxiliary/genssiReportResults.m File Reference

genssiReportResults reports the results of the analysis.

Functions

- mlhsInnerSubst< matlabtypesubstitute > genssiReportResults (matlabtypesubstitute model, matlabtypesubstitute results, matlabtypesubstitute options)

    genssiReportResults reports the results of the analysis.

7.17.1 Function Documentation

7.17.1.1 genssiReportResults()

```
mlhsInnerSubst< matlabtypesubstitute > genssiReportResults (
            matlabtypesubstitute model,
            matlabtypesubstitute results,
            matlabtypesubstitute options )
```

Parameters

model	model definition (struct)
results	results of previous steps (struct)
options	options (struct)

Return values

options	options (struct)

Definition at line 17 of file genssiReportResults.m.

Here is the caller graph for this function:

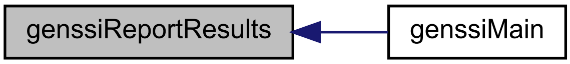

7.18 Auxiliary/genssiStructToSource.m File Reference

genSsiStructToSource converts a model definition (struct) to a source format (Matlab function file) and saves the results in the examples directory.

Functions

- noret::substitute genssiStructToSource (matlabtypesubstitute model, matlabtypesubstitute modelName)

  genSsiStructToSource converts a model definition (struct) to a source format (Matlab function file) and saves the results in the examples directory.

7.18.1 Function Documentation

7.18.1.1 genssiStructToSource()

```
noret::substitute genssiStructToSource (
            matlabtypesubstitute model,
            matlabtypesubstitute modelName )
```

Parameters

model	model definition (struct)
modelName	model name

Return values

modelName	void

Definition at line 17 of file genssiStructToSource.m.

Here is the caller graph for this function:

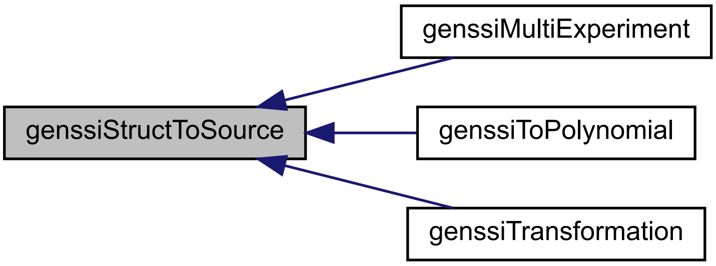

7.19 Auxiliary/genssiTableauImage.m File Reference

genssiTableauImage displays an identifiability tableau

Functions

- noret::substitute genssiTableauImage (matlabtypesubstitute figNum, matlabtypesubstitute tabMat, matlabtypesubstitute paramDisplay, matlabtypesubstitute options)

  genssiTableauImage displays an identifiability tableau

7.19.1 Function Documentation

7.19.1.1 genssiTableauImage()

```
noret::substitute genssiTableauImage (
            matlabtypesubstitute figNum,
            matlabtypesubstitute tabMat,
            matlabtypesubstitute paramDisplay,
            matlabtypesubstitute options )
```

Parameters

figNum	figure number
tabMat	matrix containing tableau
paramDisplay	parameter vector
options	options

Return values

options	void

Definition at line 17 of file genssiTableauImage.m.

Here is the caller graph for this function:

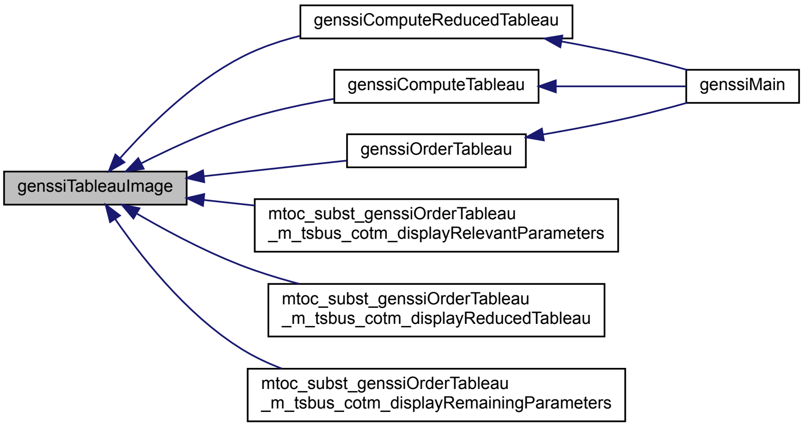

7.20 Auxiliary/genssiTransposeModel.m File Reference

genssiTransposeModel transposes all symbolic entries of the a GenSSI model. This function is necessary as different subroutines expect currently different formats. This should be corrected in future releases.

Functions

- mlhsInnerSubst< matlabtypesubstitute > genssiTransposeModel (matlabtypesubstitute model)

  genssiTransposeModel transposes all symbolic entries of the a GenSSI model. This function is necessary as different subroutines expect currently different formats. This should be corrected in future releases.

7.20.1 Function Documentation

7.20.1.1 genssiTransposeModel()

```
mlhsInnerSubst< matlabtypesubstitute > genssiTransposeModel (
            matlabtypesubstitute model )
```

Parameters

model	GenSSI model

Return values

model	GenSSI model

Definition at line 17 of file genssiTransposeModel.m.

Here is the caller graph for this function:

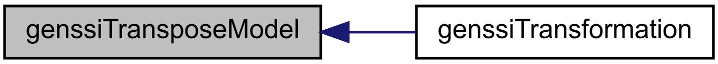

7.21 genssiMain.m File Reference

genssiMain is the main function of GenSSI. It reads a model and calls all other functions necessary for analyzing the model.

Functions

- mlhsInnerSubst< matlabtypesubstitute > genssiMain (matlabtypesubstitute modelName, matlabtypesubstitute Nder, matlabtypesubstitute Par, matlabtypesubstitute optionsIn)

  genssiMain is the main function of GenSSI. It reads a model and calls all other functions necessary for analyzing the model.

7.21.1 Function Documentation

7.21.1.1 genssiMain()

```
mlhsInnerSubst< matlabtypesubstitute > genssiMain (
            matlabtypesubstitute modelName,
            matlabtypesubstitute Nder,
            matlabtypesubstitute Par,
            matlabtypesubstitute optionsIn )
```

Parameters

modelName	the name of the model to be analyzed (a string)
Nder	number of Lie derivatives
Par	vector of parameters to be considered
optionsIn	struct of options for analysis

Return values

options	struct containing options

Definition at line 17 of file genssiMain.m.

Here is the call graph for this function:

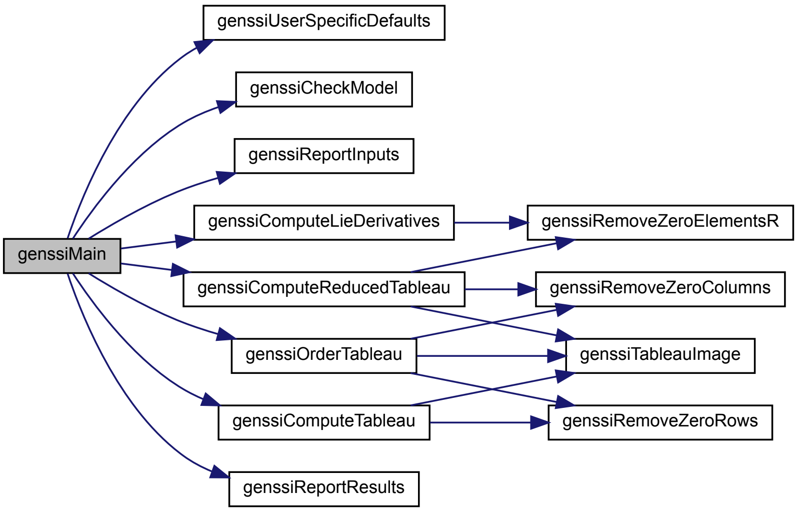

7.22 genssiMultiExperiment.m File Reference

genssiMultiExperiment converts a GenSSI model to a new GenSSI model based on a multi-experiment definition.

Functions

- noret::substitute genssiMultiExperiment (matlabtypesubstitute varargin)

  genssiMultiExperiment converts a GenSSI model to a new GenSSI model based on a multi-experiment definition.

7.22.1 Function Documentation

7.22.1.1 genssiMultiExperiment()

```
noret::substitute genssiMultiExperiment (
            matlabtypesubstitute varargin )
```

Parameters

varargin	generic input arguments<br>`genssiMultiExperiment ( modelNameIn, ExpCondName, modelNameOut )`<br>*Required Parameters for varargin:*<br>• modelNameIn the name of the input model (a string)<br>• ExpCondName the name of a file defining experimental conditions (string)<br>• modelNameOut the name of the output model (a string)

Return values

modelNameOut	void

Definition at line 17 of file genssiMultiExperiment.m.

Here is the call graph for this function:

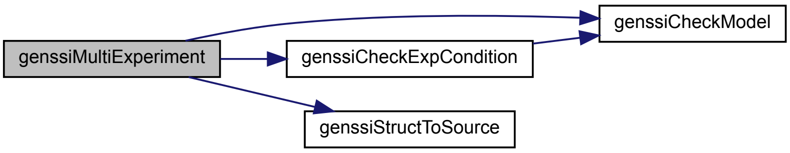

7.23 genssiStartup.m File Reference

genssiStartup adds all paths required for GenSSI. Furthermore, it generates the file genssiUserSpecificDefaults.m, providing user-specific defaults.

Functions

- noret::substitute genssiStartup ()

  genssiStartup adds all paths required for GenSSI. Furthermore, it generates the file genssiUserSpecificDefaults.m, providing user-specific defaults.

7.24 genssiToPolynomial.m File Reference

genssiToPolynomial converts a GenSSI model to polynomial form. It reads the input model, converts to polynomial form, and creates an output model as a Matlab function modelNameOut.m and as a Matlab file modelnameOut.m.

Functions

- mlhsInnerSubst< matlabtypesubstitute > genssiToPolynomial (matlabtypesubstitute modelNameIn, matlabtypesubstitute modelNameOut)

  genssiToPolynomial converts a GenSSI model to polynomial form. It reads the input model, converts to polynomial form, and creates an output model as a Matlab function modelNameOut.m and as a Matlab file modelnameOut.m.

7.24.1 Function Documentation

7.24.1.1 genssiToPolynomial()

```
mlhsInnerSubst< matlabtypesubstitute > genssiToPolynomial (
            matlabtypesubstitute modelNameIn,
            matlabtypesubstitute modelNameOut )
```

Parameters

modelNameIn	the name of the input model (a string)
modelNameOut	the name of the output model (a string)

Return values

modelNameOut	void

Definition at line 17 of file genssiToPolynomial.m.

Here is the call graph for this function:

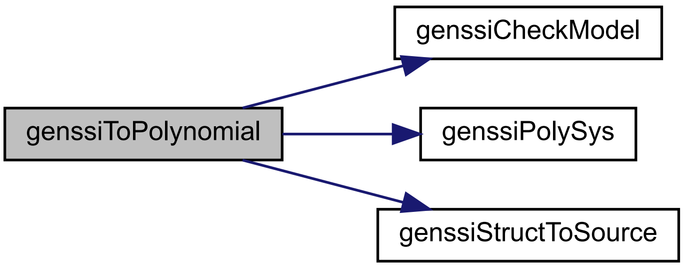

7.25 genssiTransformation.m File Reference

genssiTransformation converts a GenSSI model to a new GenSSI model based on a transformation definition.

Functions

- noret::substitute genssiTransformation (matlabtypesubstitute varargin)
  genssiTransformation converts a GenSSI model to a new GenSSI model based on a transformation definition.

7.25.1 Function Documentation

7.25.1.1 genssiTransformation()

```
noret::substitute genssiTransformation (
            matlabtypesubstitute varargin )
```

Parameters

varargin	generic input arguments<br>`genssiTransformation ( modelNameIn, transDef, modelNameOut )`<br>*Required Parameters for varargin:*<br>• modelNameIn the name of the input model (a string)<br>• transDef the name of a transformation definition file (string)<br>• modelNameOut the name of the output model (a string)

Return values

modelNameOut	void

Definition at line 17 of file genssiTransformation.m.

Here is the call graph for this function:

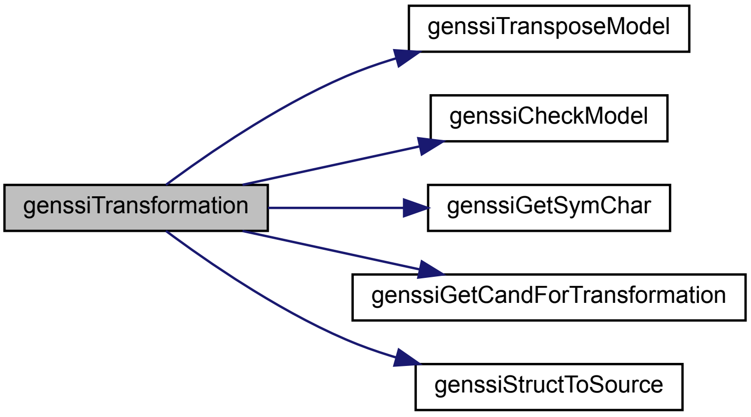

7.26 genssiUserSpecificDefaults.m File Reference

genssiUserSpecificDefaults defines ths user-specific defaults. This file can be altered by the user to personalize the settings.

Functions

- mlhsInnerSubst< matlabtypesubstitute > genssiUserSpecificDefaults (matlabtypesubstitute optionName)
  genssiUserSpecificDefaults defines ths user-specific defaults. This file can be altered by the user to personalize the settings.

7.26.1 Function Documentation

7.26.1.1 genssiUserSpecificDefaults()

```
mlhsInnerSubst< matlabtypesubstitute > genssiUserSpecificDefaults (
            matlabtypesubstitute optionName )
```

Parameters

optionName	name of option

Return values

default	defualt values of option

Definition at line 17 of file genssiUserSpecificDefaults.m.

Here is the caller graph for this function:

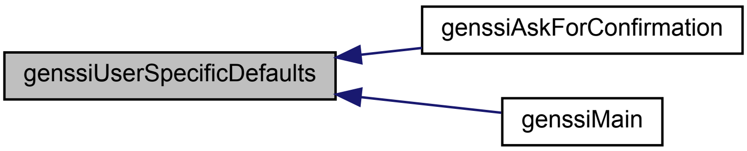

7.27 SBMLimporter/computeBracketLevel.m File Reference

Compute the bracket level for the input string cstr. The bracket level is computed for every char in cstr and indicates how many brackets have been opened up to this point. The bracket level is useful to parse the arguments of functions in cstr. For this purpose functions will have the same bracket level as the opening bracket of the corresponding function call.

Functions

- mlhsInnerSubst<::*int > computeBracketLevel (::*char cstr)

  Compute the bracket level for the input string cstr. The bracket level is computed for every char in cstr and indicates how many brackets have been opened up to this point. The bracket level is useful to parse the arguments of functions in cstr. For this purpose functions will have the same bracket level as the opening bracket of the corresponding function call.

7.27.1 Function Documentation

7.27.1.1 computeBracketLevel()

```
mlhsInnerSubst<::*int > computeBracketLevel (
              ::*char cstr )
```

Parameters

cstr	input string

Return values

brl	bracket levels

Definition at line 17 of file computeBracketLevel.m.

Here is the caller graph for this function:

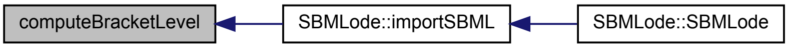

Index

amiciStructToSource
    amiciStructToSource.m, 23
amiciStructToSource.m
    amiciStructToSource, 23
Auxiliary/amiciStructToSource.m, 23
Auxiliary/genssiAskForConfirmation.m, 24
Auxiliary/genssiCheckExpCondition.m, 25
Auxiliary/genssiCheckModel.m, 26
Auxiliary/genssiComputeLieDerivatives.m, 27
Auxiliary/genssiComputeReducedTableau.m, 29
Auxiliary/genssiComputeTableau.m, 30
Auxiliary/genssiGetCandForTransformation.m, 31
Auxiliary/genssiGetSymChar.m, 32
Auxiliary/genssiOrderTableau.m, 33
Auxiliary/genssiPolySys.m, 35
Auxiliary/genssiRemoveZeroColumns.m, 36
Auxiliary/genssiRemoveZeroElementsC.m, 37
Auxiliary/genssiRemoveZeroElementsR.m, 38
Auxiliary/genssiRemoveZeroRows.m, 39
Auxiliary/genssiReportInputs.m, 40
Auxiliary/genssiReportResults.m, 41
Auxiliary/genssiStructToSource.m, 42
Auxiliary/genssiTableauImage.m, 43
Auxiliary/genssiTransposeModel.m, 44

bolus
    SBMLode, 22

checkODE
    SBMLode, 18
compartment
    SBMLode, 20
computeBracketLevel
    computeBracketLevel.m, 51
computeBracketLevel.m
    computeBracketLevel, 51
condition
    SBMLode, 21
constant
    SBMLode, 20

flux
    SBMLode, 21
funarg
    SBMLode, 22
funmath
    SBMLode, 22

genssiAskForConfirmation
    genssiAskForConfirmation.m, 24
genssiAskForConfirmation.m
    genssiAskForConfirmation, 24
genssiCheckExpCondition
    genssiCheckExpCondition.m, 25
genssiCheckExpCondition.m
    genssiCheckExpCondition, 25
genssiCheckModel
    genssiCheckModel.m, 26
genssiCheckModel.m
    genssiCheckModel, 26
genssiComputeLieDerivatives
    genssiComputeLieDerivatives.m, 27
genssiComputeLieDerivatives.m
    genssiComputeLieDerivatives, 27
    mtoc_subst_genssiComputeLieDerivatives_m_tsbus_cotm_jacRank,
        28
genssiComputeReducedTableau
    genssiComputeReducedTableau.m, 29
genssiComputeReducedTableau.m
    genssiComputeReducedTableau, 29
genssiComputeTableau
    genssiComputeTableau.m, 30
genssiComputeTableau.m
    genssiComputeTableau, 30
genssiGetCandForTransformation
    genssiGetCandForTransformation.m, 32
genssiGetCandForTransformation.m
    genssiGetCandForTransformation, 32
genssiGetSymChar
    genssiGetSymChar.m, 32
genssiGetSymChar.m
    genssiGetSymChar, 32
genssiMain
    genssiMain.m, 45
genssiMain.m, 45
    genssiMain, 45
genssiMultiExperiment
    genssiMultiExperiment.m, 46
genssiMultiExperiment.m, 46
    genssiMultiExperiment, 46
genssiOrderTableau
    genssiOrderTableau.m, 34
genssiOrderTableau.m
    genssiOrderTableau, 34
genssiPolySys
    genssiPolySys.m, 35
genssiPolySys.m
    genssiPolySys, 35
genssiRemoveZeroColumns
    genssiRemoveZeroColumns.m, 37
genssiRemoveZeroColumns.m
    genssiRemoveZeroColumns, 37
genssiRemoveZeroElementsC.m
    genssiRemoveZeroElementsC, 37
genssiRemoveZeroElementsR.m
    genssiRemoveZeroElementsR, 38
genssiRemoveZeroElementsC
    genssiRemoveZeroElementsC.m, 37
genssiRemoveZeroElementsR
    genssiRemoveZeroElementsR.m, 38
genssiRemoveZeroRows

genssiRemoveZeroRows.m, 39
genssiRemoveZeroRows.m
genssiRemoveZeroRows, 39
genssiReportInputs
genssiReportInputs.m, 40
genssiReportInputs.m
genssiReportInputs, 40
genssiReportResults
genssiReportResults.m, 41
genssiReportResults.m
genssiReportResults, 41
genssiStartup.m, 47
genssiStructToSource
genssiStructToSource.m, 42
genssiStructToSource.m
genssiStructToSource, 42
genssiTableauImage
genssiTableauImage.m, 43
genssiTableauImage.m
genssiTableauImage, 43
genssiToPolynomial
genssiToPolynomial.m, 48
genssiToPolynomial.m, 47
genssiToPolynomial, 48
genssiTransformation
genssiTransformation.m, 49
genssiTransformation.m, 48
genssiTransformation, 49
genssiTransposeModel
genssiTransposeModel.m, 44
genssiTransposeModel.m
genssiTransposeModel, 44
genssiUserSpecificDefaults
genssiUserSpecificDefaults.m, 50
genssiUserSpecificDefaults.m, 50
genssiUserSpecificDefaults, 50

importSBML
SBMLode, 17
initState
SBMLode, 21

knom
SBMLode, 23
kvolume
SBMLode, 21

mtoc_subst_genssiComputeLieDerivatives_m_tsbus_cotm_jacRank
genssiComputeLieDerivatives.m, 28

observable
SBMLode, 19
observable_name
SBMLode, 19

param
SBMLode, 19
parameter
SBMLode, 20
pnom
SBMLode, 23

reaction
SBMLode, 20

SBMLimporter/computeBracketLevel.m, 50
SBMLode, 14
bolus, 22
checkODE, 18
compartment, 20
condition, 21
constant, 20
flux, 21
funarg, 22
funmath, 22
importSBML, 17
initState, 21
knom, 23
kvolume, 21
observable, 19
observable_name, 19
param, 19
parameter, 20
pnom, 23
reaction, 20
SBMLode, 16
state, 19
stochiometry, 21
time_symbol, 23
trigger, 22
volume, 20
writeAMICI, 18
xdot, 22
state
SBMLode, 19
stochiometry
SBMLode, 21

time_symbol
SBMLode, 23
trigger
SBMLode, 22

volume
SBMLode, 20

writeAMICI
SBMLode, 18

xdot
SBMLode, 22
